# A Quantitative Study of the Division Cycle of Caulobacter crescentus Stalked Cells

**DOI:** 10.1371/journal.pcbi.0040009

**Published:** 2008-01-25

**Authors:** Shenghua Li, Paul Brazhnik, Bruno Sobral, John J Tyson

**Affiliations:** 1 Department of Biological Sciences, Virginia Polytechnic Institute and State University, Blacksburg, Virginia, United States of America; 2 Virginia Bioinformatics Institute, Virginia Polytechnic Institute and State University, Blacksburg, Virginia, United States of America; Lawrence Berkeley National Laboratory, United States of America

## Abstract

Progression of a cell through the division cycle is tightly controlled at different steps to ensure the integrity of genome replication and partitioning to daughter cells. From published experimental evidence, we propose a molecular mechanism for control of the cell division cycle in Caulobacter crescentus. The mechanism, which is based on the synthesis and degradation of three “master regulator” proteins (CtrA, GcrA, and DnaA), is converted into a quantitative model, in order to study the temporal dynamics of these and other cell cycle proteins. The model accounts for important details of the physiology, biochemistry, and genetics of cell cycle control in stalked C. crescentus cell. It reproduces protein time courses in wild-type cells, mimics correctly the phenotypes of many mutant strains, and predicts the phenotypes of currently uncharacterized mutants. Since many of the proteins involved in regulating the cell cycle of C. crescentus are conserved among many genera of α-proteobacteria, the proposed mechanism may be applicable to other species of importance in agriculture and medicine.

## Author Summary

The cell cycle is the sequence of events by which a growing cell replicates all its components and divides them more or less evenly between two daughter cells. The timing and spatial organization of these events are controlled by gene–protein interaction networks of great complexity. A challenge for computational biology is to build realistic, accurate, predictive mathematical models of these control systems in a variety of organisms, both eukaryotes and prokaryotes. To this end, we present a model of a portion of the molecular network controlling DNA synthesis, cell cycle–related gene expression, DNA methylation, and cell division in stalked cells of the α-proteobacterium Caulobacter crescentus. The model is formulated in terms of nonlinear ordinary differential equations for the major cell cycle regulatory proteins in *Caulobacter*: CtrA, GcrA, DnaA, CcrM, and DivK. Kinetic rate constants are estimated, and the model is tested against available experimental observations on wild-type and mutant cells. The model is viewed as a starting point for more comprehensive models of the future that will account, in addition, for the spatial asymmetry of *Caulobacter* reproduction (swarmer cells as well as stalked cells), the correlation of cell growth and division, and cell cycle checkpoints.

## Introduction

The events of the cell division cycle must be carried out in a coordinated fashion. Coordination is maintained by underlying molecular regulatory systems of great complexity. Intensive studies of these protein interaction networks by mathematical modeling have assisted our understanding of cell cycle regulation in yeasts [1–5], frog eggs [6–8], and even mammalian cells [9,10]. In addition to reproducing large amounts of experimental data, these models have made successful predictions and guided further experimental studies [11–14].

Although progress in understanding cell cycle regulation in bacteria has lagged behind eukaryotes, the recent discovery of master regulatory proteins, CtrA and GcrA, in Caulobacter crescentus [15,16] allowed us to propose a closed loop of signaling events controlling the cell cycle in this bacterium [17]. Central to this proposal is a CtrA-based bistable switch [17] that can be driven to the on state by GcrA and to the off state by cell division. This simple model, though providing some insight into the logic of cell cycle regulation in *Caulobacter*, neglected many important aspects of the control system. In this paper, we add more genetic and molecular details to the Brazhnik-Tyson model in order to build a computational model of sufficient accuracy to account for the phenotypes of many mutant strains, both well-characterized and yet-to-be-studied strains. We have incorporated a third important regulatory protein, DnaA [18,19], and the effects of DNA methylation on gene expression. In particular, as the DNA replication fork progresses along the bacterial chromosome, it may turn on the expression of an inactive (fully methylated) gene by creating a pair of hemimethylated genes (old strand methylated, new strand unmethylated). These genes are returned to their fully methylated state by CcrM, a methyltransferase whose synthesis is induced by CtrA late in the cycle. (Some genes are active in the methylated state and inactive in the hemimethylated state.) Genes that are regulated in this fashion can be turned on/off in a strict temporal order, according to their location on the chromosome. The role of DNA methylation in governing progression through the *Caulobacter* cell cycle has recently been emphasized by Collier et al. [20].

At this stage of the model, the regulation of CtrA proteolysis has been incorporated in a simplistic way, concentrating on the phosphorylation of DivK in the stalked cell compartment at cell division and ignoring (for now) the roles of other proteins, such as RcdA, CpdR, and ClpXP [21]. In addition, we do not attempt to model the control of CtrA activity by phosphorylation [22,23], nor do we take into account explicitly the spatial localization of proteins [24,25]. These aspects of the control system are reserved for a later version of the model.

### A Consensus Picture of Cell Cycle Controls in C. crescentus

**Figure 1:**
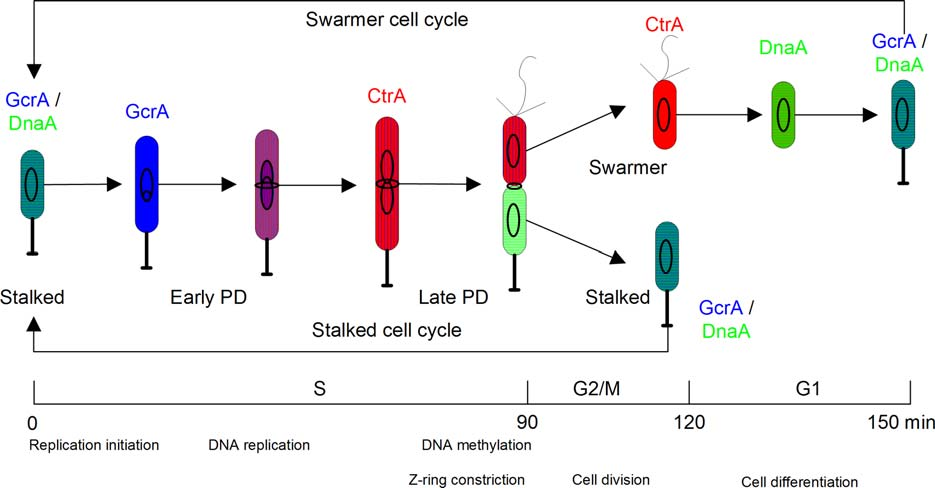
Physiology of the Cell Division Cycle in Caulobacter crescentus Three cell cycle phases can be distinguished in a swarmer cell: a growth and differentiation (G1) phase that lasts approximately 30 min, a DNA synthesis (S) phase that takes approximately 90 min, and a cell division (G2/M) phase, lasting approximately 30 min, that culminates in the separation of mother (stalked) and daughter (swarmer) cells. The stalked cell cycle lacks G1 phase. The color scheme denotes protein variations through the cell division cycle: GcrA (blue), CtrA (red), DnaA (green). The θ-like structure denotes replicating DNA. The ring in the middle of the cell indicates Z-ring formation and constriction, leading to cell separation (cytokinesis). PD, predivisional.

**Figure 2:**
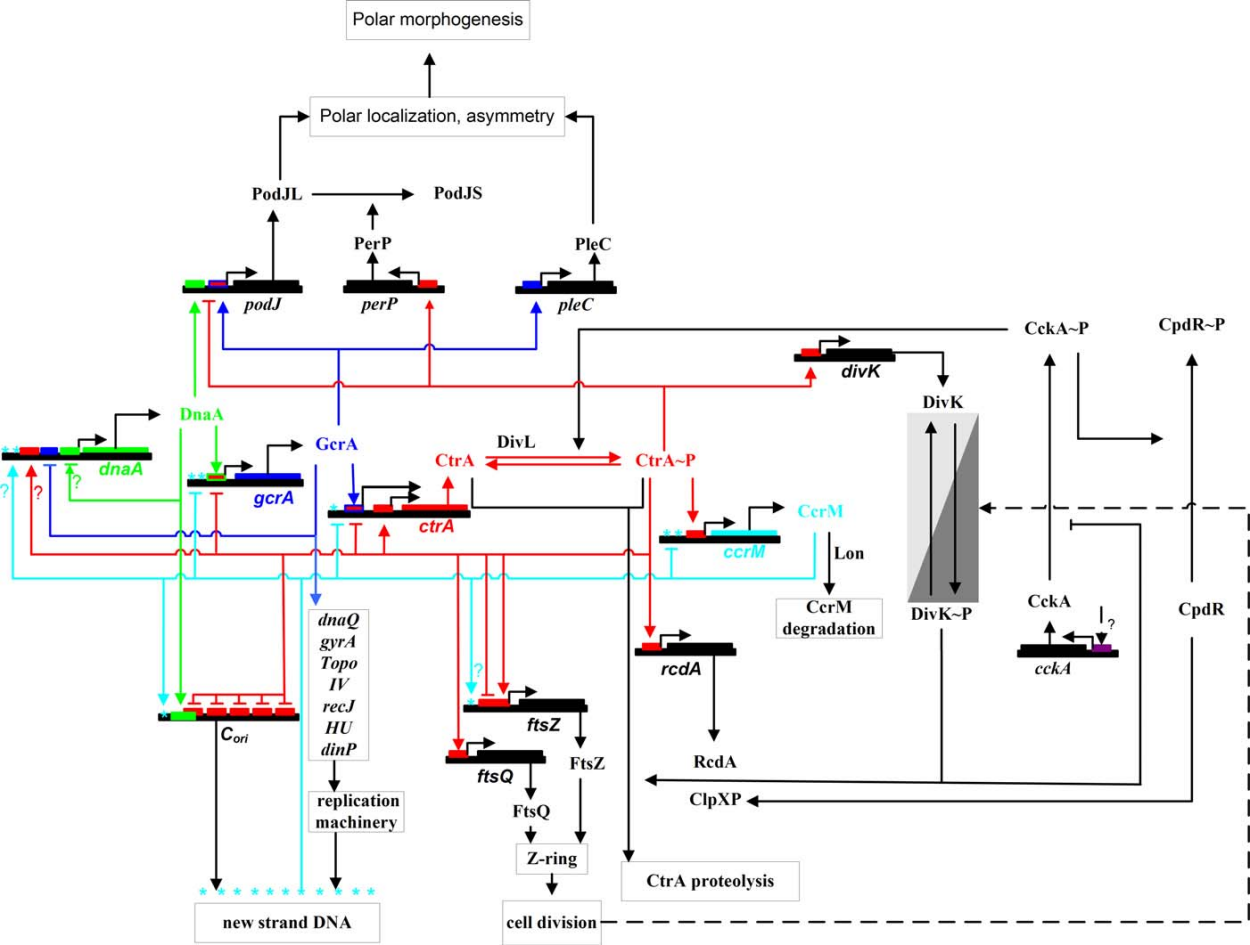
Known Cell Cycle Genes in Caulobacter crescentus (Adapted from [77]) Regulation of genes by CtrA is shown in red, by GcrA in blue, by DnaA in green, and by CcrM in cyan. The cyan stars indicate those genes whose transcription is regulated by DNA methylation. The CtrA-driven up-regulation of the *dnaA* gene (red line with ? mark, left) is suggested by microarray data [32]. DnaA self-regulation (blue line with ? mark, left) is proposed from the fact that the *dnaA* promoter has DnaA boxes [55].

C. crescentus is a dimorphic bacterium that inhabits freshwater, seawater, and soils, where it plays an important role in global carbon cycling by mineralizing dissolved organic materials [26]. C. crescentus normally undergoes an asymmetric cell division resulting in two different progeny cells (Figure 1): a motile, flagellated swarmer cell and a sessile stalked cell [22,23,27]. The nascent stalked cell then enters immediately into a new round of cell division and produces, about 90–120 min later, a new swarmer cell. The nascent swarmer cell swims around for 30–45 min before it differentiates into a stalked cell and initiates the DNA replication–division cycle. In this paper, we restrict our attention to the division cycle of stalked cells. Figure 2 depicts central elements of the cell division regulatory network in *C. crescentus.*

### CtrA and GcrA: Master Regulatory Proteins

Caulobacter crescentus has 3,767 protein-encoding genes [28], of which 553 are cell cycle regulated [29]. Two master-regulator proteins control more than 25% of cell cycle–regulated genes: the transcription factor CtrA [30] directly regulates 95 genes (including *divK*, *ccrM*, *podJ*, *pleC*, *ftsZ*, and *ftsQ*) [31,32], whereas GcrA controls 49 genes [15,29,32]. There is also weak evidence from microarray data [32] that CtrA can up-regulate *dnaA*. In addition, DNA synthesis in C. crescentus is under direct control by CtrA [33–35], which binds to the origin of DNA replication and inhibits initiation of DNA synthesis [30].

CtrA is present at a high level in swarmer cells, whereas in stalked cells, it changes from low to high level during the cell cycle [15,36,37]. The abundance and activity of the CtrA protein is regulated through gene transcription, protein degradation, and phosphorylation.

Expression of *ctrA* is under control of two promoters, *ctrA* P1 and *ctrA* P2 [31,36,38]. The weaker *ctrA* P1 promoter is activated in the early stalked cell (∼35 min after the initiation of DNA replication [39,40]) by GcrA protein [15] and inhibited by high levels of CtrA itself [36]. The stronger *ctrA* P2 promoter is activated later, in predivisional cells, by the CtrA protein itself [36]. In addition, the *ctrA* P1 promoter is only activated from a new strand of hemimethylated DNA [31,40]. The *ctrA* P2 promoter is not active in swarmer cells, even though these cells have high levels of CtrA [36]. Furthermore, expression from *ctrA* P2 is inhibited in predivisional cells by conditions that inhibit DNA replication [41]. These facts indicate that *ctrA* P2 has regulators other than CtrA itself [36].

Proteolysis of CtrA (and CtrA∼P) is significantly accelerated by the phosphorylated form of DivK protein, DivK∼P, via the ClpXP protease pathway [42], or with the help of some other (as yet unknown) histidine phosphotransferases [43]. Recently, RcdA and CpdR proteins have been reported to be involved in CtrA degradation in combination with ClpXP [44,45]. When this proteolysis pathway is activated, the half-life of CtrA in vivo is 5 min or less [38].

CtrA is active when phosphorylated [46,47], a reaction carried out by a histidine kinase, CckA [46,48], and a histidine phosphotransferase, ChpT [49]. In addition, CtrA is also phosphorylated by a tyrosine kinase, DivL [50]. CtrA is rapidly dephosphorylated in vivo. The activity of CckA was shown recently to be down-regulated by a DivK∼P [44,45,49], thereby linking the phosphorylation and proteolysis pathways of CtrA. But otherwise, how the kinase and phosphatase reactions are regulated to control the fraction of active CtrA is poorly understood.

GcrA is an activator of components of the replisome and of the segregation machinery [15], and also regulates genes such as *ctrA*, *pleC*, and *podJ* [15,19]. GcrA protein concentration varies through the cell division cycle, peaking early in the cycle in stalked cells and reaching its minimum in a swarmer cell, after cell division. The DNA replication-initiating protein, DnaA, is required for *gcrA* expression [18]. In addition, transcription of *gcrA* is repressed by the CtrA protein [15].

### DNA Replication

DNA replication proceeds in three phases: initiation, elongation, and termination. The origin of DNA replication (C_ori_) in C. crescentus has one potential binding site for DnaA, a protein involved in initiating DNA synthesis [51]. The DnaA binding site partially overlaps with five CtrA binding sites in C_ori_ [33–35]. CtrA represses initiation of DNA replication [30]. Thus, DNA replication is only initiated when DnaA level is high and CtrA level is low. In addition, DNA replication cannot be re-initiated until the origin stie has been fully methylated [52,53]. These conditions prevail during the swarmer-to-stalked cell transition, and just after division in the stalked cell compartment [34]. Once initiated, DNA synthesis continues bidirectionally along the circular chromosome, with an average speed of ∼20.5 kb/min in minimal broth, finishing in the late predivisional cell [54]. Elongation of newly replicating DNA strands requires a complex machinery, many components of which are under GcrA control [15].

### DNA Methylation by CcrM

Several cell cycle–related genes (*ctrA*, *gcrA*, *dnaA*, *ftsZ*, and *ccrM*) have GANTC methylation sites in their promoters [19,31,40,52,53,55,56]. Hence, the expression of these genes may be sensitive to the methylation state of the promoter. DNA replication transforms a fully methylated gene (both strands methylated) into a pair of hemimethylated genes (only one strand methylated). At some later time, the unmethylated strands become methylated by the action of CcrM to return the genes to the fully methylated state [53]. These methylation transitions may affect the expression of cell cycle–related genes [53]. Methylation of C_ori_ is also necessary for initiating a new round of DNA synthesis [34]. These methylation effects provide feedback from the progression of DNA replication to the cell cycle control system.

In C. crescentus and other α-proteobacteria, CcrM is the methyltransferase that accounts for methylation of newly synthesized DNA strands. *ccrM* transcription is activated by CtrA only from a hemimethylated chromosome for about 20 min, in a late predivisional cell (its expression peaks at ∼105 min in the 150-min swarmer cell cycle) [57]. Lon protease is required for CcrM degradation [58]. The half-life of CcrM is less than 10 min in vivo [39]. Thus, CcrM activity is limited to a short portion of the predivisional cell phase, just before cell division.

### The Septal or Z-Ring

The multicomponent Z-ring organelle, which forms and constricts at the mid-cell plane, plays an important role in compartmentation of the predivisional cell and its subsequent division [27]. Compartmentation lasts about 20 min [59]. After the late predivisional cell is divided into two progeny cells, the Z-ring is disassembled and degraded.

The Fts proteins (FtsZ, FtsQ, FtsA, and FtsW) have been identified as crucial elements of the Z-ring. *ftsZ* expression is positively and negatively regulated by CtrA [29,60], and it may also by regulated by DNA methylation since the *ftsZ* promoter has a methylation site [40,53]. The *ftsQ* gene is expressed only after CtrA-mediated activation in the late predivisional cell [41]. The FtsQ protein localizes predominantly to the mid-cell plane of the predivisional cell, consistently with the appearance of the Z-ring [61,62]. The FtsA protein exhibits the time course similar to FtsQ [61].

### Polar Distribution of DivK and DivK∼P

*divK* transcription is activated by CtrA in late predivisional cells, which results in a slight elevation of DivK protein, otherwise present throughout the cell cycle at a nearly constant level [42,63]. The total amount of DivK∼P, the form that promotes CtrA degradation, does not appear to undergo dramatic changes during the cell cycle. It is 50% ± 20% lower in swarmer cells than in predivisional cells [63]. However, DivK and DivK∼P are dynamically localized during the cell division cycle [63–68]. Membrane-bound proteins DivJ and PleC, which localize at stalked and flagellated cell poles, respectively, regulate this process [64,65] by having opposite effects on DivK phosphorylation. DivJ is a kinase that continuously phosphorylates DivK at the stalked cell pole, and PleC promotes the continuous dephosphorylation of DivK∼P at the flagellated cell pole [64,67]. Hence, opposing gradients of DivK and DivK∼P are established between the two cell poles. Full constriction of the Z-ring disrupts the diffusion of DivK between the two poles [59,64]. As a result, DivK∼P accumulates in the nascent stalked cell compartment and unphosphorylated DivK accumulates in the nascent swarmer cell compartment. High DivK∼P promotes CtrA degradation in the stalked cell compartment [42,43], whereas high CtrA is maintained in the swarmer cell compartment [16]. The nonuniform distribution of DivK and DivK∼P, and their corresponding effects on CtrA degradation, contribute largely to the different developmental programs of swarmer and stalked cells in C. crescentus. In addition, recent investigations indicate that CtrA phosphorylation is also at least partially under the control of DivK∼P (as mentioned above), which shows that DivK∼P not only controls the stability of CtrA, but also its activity [44,45].

## Results

**Figure 3:**
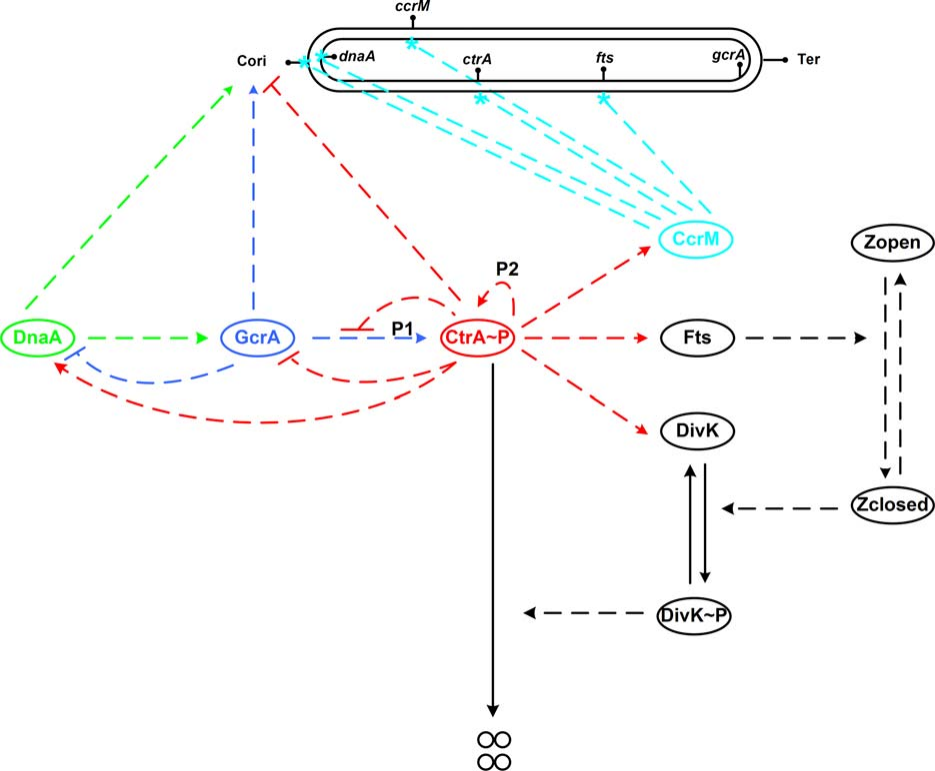
Wiring Diagram of the DNA Replication-Driven Model The double-stranded closed curve at the top represents DNA. C_ori_ is the origin of DNA replication and *Ter* stands for the termination site. All proteins (ovals) are assumed to be produced and degraded at specific rates. Only the degradation of CtrA is shown on the diagram (four small circles indicate products of CtrA degradation), in order to indicate how this step is regulated by closure of the Z-ring (Zclosed) and subsequent phosphorylation of DivK. Dashed lines denote regulatory effects among the components. DNA methylation sites on genes are marked by cyan stars.

Using Figure 2, we create a wiring diagram (Figure 3) of the molecular interactions that we deem to be most important for regulation of the division cycle in stalked cells. The diagram is then converted into a mathematical model (Table 1), as described in Materials and Methods. Full details of the model can be found on our Web site (http://mpf.biol.vt.edu/research/caulobacter/pp/), including machine-readable versions of the model (for MATLAB and SBML) and an online simulator.

**Table 1:**
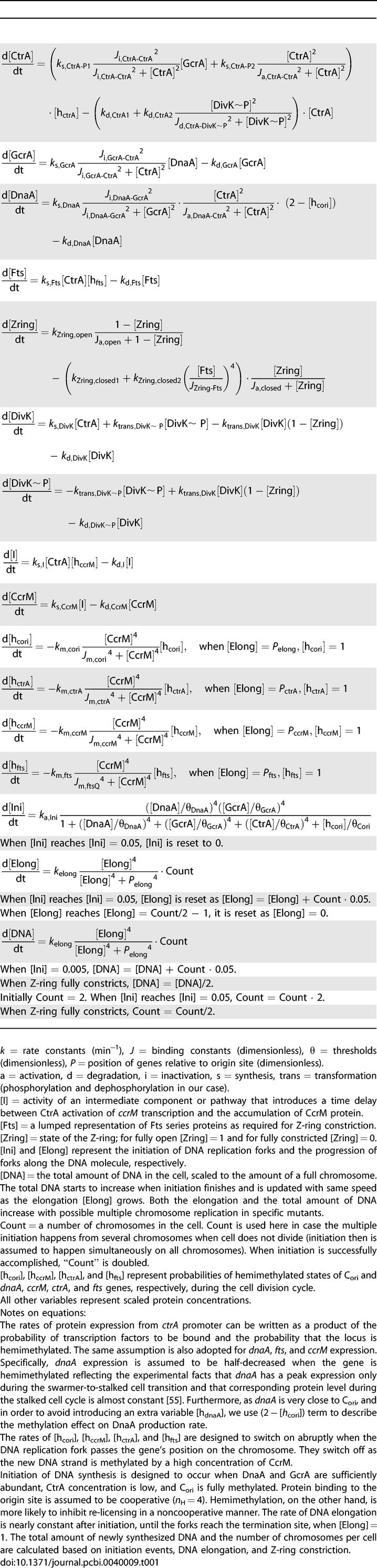
Equations of the Model

### The Model Accurately Describes Protein Expression Patterns during the Division Cycle of Wild-Type Cells

**Figure 4:**
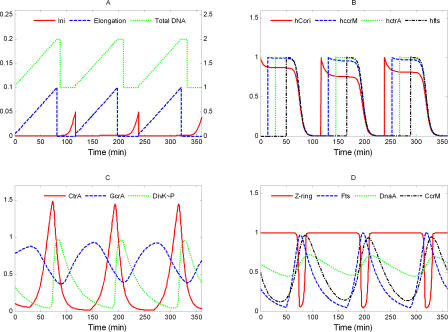
Simulated Variations of Model State Variables during the Wild-Type Cell Cycle Here and in subsequent figures, the simulation begins when the initiation of DNA replication has completed. Three cell cycles are presented. (A) Here and in subsequent figures, the scale for [Ini] is on the left and the scales for [Elong] and [DNA] are on the right. DNA replication (green curve) takes 90 min, as observed [54]. Initiation (red) is elevated only for a short period of time to start DNA replication. The DNA curve (green) differs from the Elongation curve (blue) only by an additive constant equal to the number of full chromosomes in the cell. (B) The methylation states of C_ori_ and of three genes. As replication starts, C_ori_(red) is hemimethylated (*h*_Cori_ = 1), and *ccrM* (blue), *ctrA* (green), *fts* (black), and *dnaA* (unpublished data) are fully methylated (*h*_.._ = 0). As the replication forks proceed along the chromosome, these genes become hemimethylated in order, according to their positions on the chromosome. At the end of the cycle, when CcrM is active, all these sites become fully methylated. (C) Early in the cycle, GcrA (blue) is increasing and triggers production of CtrA (red). When CtrA is high, it represses synthesis of GcrA and activates its own degradation by up-regulating DivK∼P (green). (D) When a cell enters the predivisional phase, high CtrA activates the expression of *fts* genes (blue), which promote formation and constriction of the Z-ring (red). DnaA (green) and CcrM (black) are required for DNA initiation (A) and DNA methylation (B), respectively.

To simulate the molecular regulation of a wild-type stalked-cell division cycle, we solve the equations in Table 1 subject to the parameter values and initial conditions in Tables 2 and 3. Figure 4 illustrates how scaled protein concentrations and other variables of the model change during repetitive cycling of a stalked cell. The duration of a wild-type stalked-cell division cycle in our simulations is 120 min (∼90 min for S phase and ∼30 min for G2/M phase), as typically observed in experiments [22,23,59].

**Table 2:**
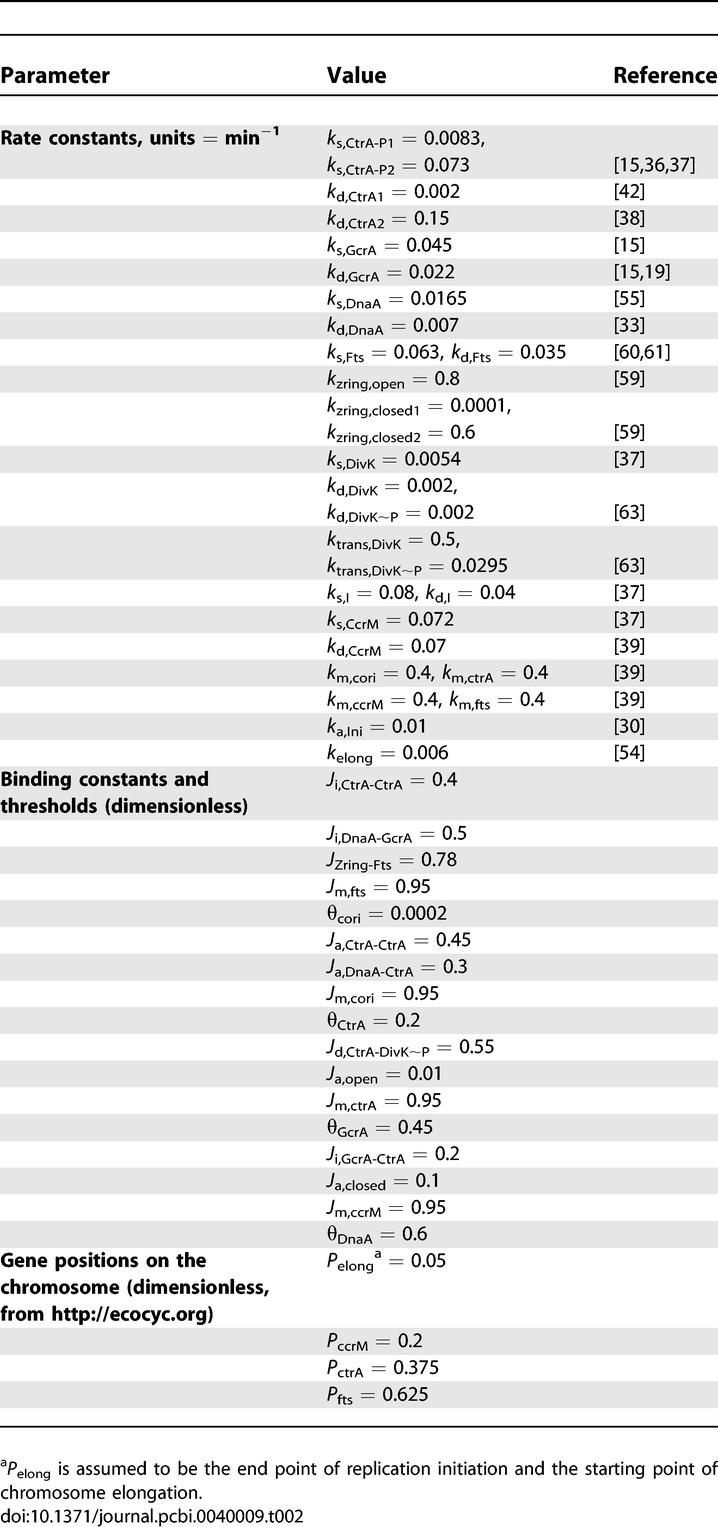
Basal Parameter Values for the Wild-Type Stalked-Cell Division Cycle

**Table 3:**
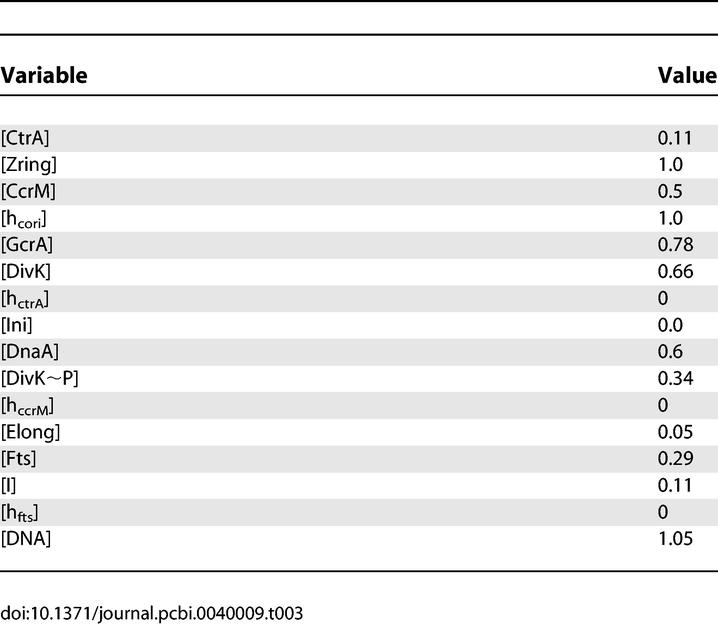
Initial Values of Model Variables, for a Newborn, Wild-Type Stalked Cell

The main physiological events of the division cycle can be traced back to characteristic signatures of protein expression, as described in the Introduction. The division cycle starts with initiation of DNA replication (Figure 4A) from a fully methylated origin site by elevated DnaA, when CtrA is low and GcrA is sufficiently high (to induce production of required components of the replication machinery) (Figure 4C and 4D). Immediately after DNA replication starts, C_ori_ is hemimethylated.

As DNA synthesis progresses, certain genetic loci become hemimethylated in order along the chromosome (Figure 4B). Consequently, the regulatory proteins are produced and reach their peak concentrations sequentially. By contrast, *dnaA* expression seems to be activated by full methylation [55], so its expression rate declines immediately after DNA replication starts. The effect of methylation on *dnaA* expression is minor compared to the regulatory signals coming from GcrA and CtrA. When the replication fork passes the *ccrM* locus, the gene becomes available for transcription, but is not immediately expressed, because CtrA level is low. In a predivisional cell, at approximately 35 min after start of DNA replication, the replication fork passes the *ctrA* gene (Figure 4B), and its expression is immediately activated by GcrA (Figure 4C) and then further up-regulated by CtrA itself. Later on, when CtrA level becomes high, expression of the *ccrM* gene and, later, hemimethylated *fts* genes (at ∼65 min), are expressed by the activation from high-level CtrA (Figure 4D).

High CtrA down-regulates *gcrA* expression. When DNA replication is finished, the new DNA strands are methylated by elevated CcrM in about 20 min. DNA methylation shuts down production of CtrA, CcrM, and Fts proteins. Meanwhile, elevated Fts proteins promote Z-ring formation and constriction (Figure 4D), which separates the predivisional cell into two compartments, thereby restricting access of DivK and DivK∼P to only one of the old poles of the cell. As a result, in the stalked cell compartment, most DivK is converted into DivK∼P, accelerating CtrA proteolysis there (Figure 4C). In a nascent stalked cell, low CtrA concentration releases *gcrA* expression, and GcrA protein level rises. Then, low CtrA, high GcrA, and high DnaA drive the nascent stalked cell into a new round of DNA synthesis from the fully methylated chromosome. These computed properties of the model agree reasonably well with what is known (or expected) about cell cycle progression in C. crescentus.

**Figure 5:**
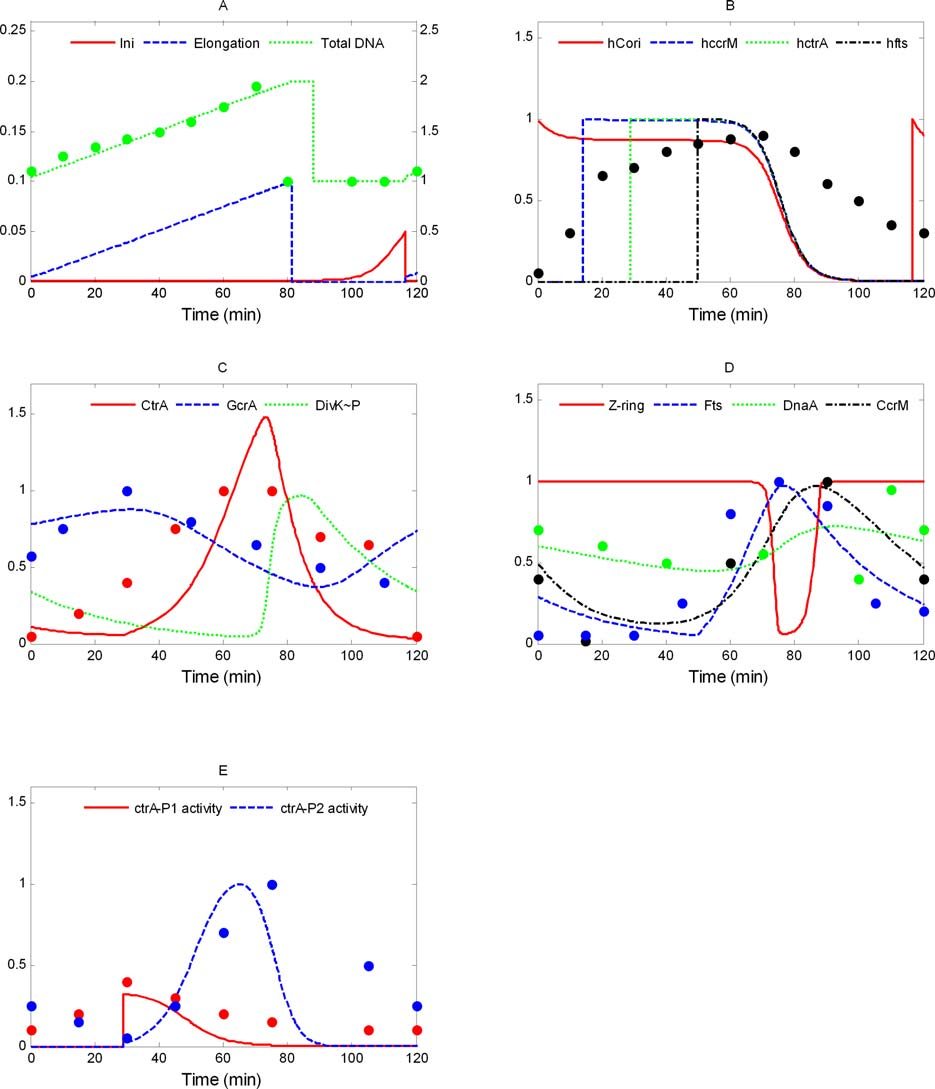
Comparison of Simulated Protein Time Profiles and DNA Accumulation (Curves) with Experimental Data (Circles) (A) The simulated total DNA (green), Elongation (blue), and Initiation (red) variables. The experimental data for total DNA are taken from Figure 4 in [54]. (B) Curves are simulated probabilities of hemimethylated states of C_ori_ and three genes. The appearance of hemimethylated gene sites in our simulation reflects the nearly linear growth of overall DNA-hemimethylation observed experimentally (data points [black] from Figure 3 in [39]). The peak of hemimethylation (∼75 min) in our simulation agrees well with experimental observation. (C) Time courses of GcrA (blue) and CtrA (red) are compared against experimental data from Figure 3 in [15] and Figure 1 in [37], respectively. Fluctuations of DivK∼P (green) are discussed in the text. (D) The generalized Fts protein time course (blue) is compared to the measured profile of FtsQ from Figure 2 in [61]. Our simulation of Z-ring constriction (red curve) is consistent with the approximately 20-min closure time reported in [59], Figure 2. CcrM (black) accumulates to high level only in the late predivisional cell, in agreement with the data (black) in [37], Figure 2. DnaA data (green) from Figure 5 in [55] do not show significant variations during the stalked cell cycle, consistent with our simulation (green curve). (E) The relative activities of *ctrA* promoters, *ctrA-P1* (red) and *ctrA-P2* (blue), as reported in Figure 4A in [40], compares well with our simulation (red and blue curves).

In Figure 5, we compare our simulation with experimental data. The data, collected from literature, were obtained by different research group with various experimental techniques. In most cases, experimental uncertainties of the data were not reported, but it is reasonable to assume that the error bounds are quite generous. Therefore, based on a visual comparison, we conclude that our model is in reasonable agreement with experimental observations.

The only serious objection that might be raised is to our simulation of DivK∼P (Figure 5C, green curve), which increases rapidly in the stalked-cell compartment after the Z-ring closes and DivK∼P is cut off from its phosphatase at the swarmer cell pole. Jacobs et al. [62] reported roughly constant levels of DivK∼P in predivisional stalked cells, i.e., until just before Z-ring constriction, and significant differences of DivK∼P levels between stalked cells and swarmer cells. Our waveform for DivK∼P is consistent with this report and predicts that there should be a distinct peak of DivK phosphorylation in the stalked cell compartment at the end of the division cycle. This peak seems to be an inevitable consequence of the current belief that, upon Z-ring constriction, DivK becomes dephosphorylated in the swarmer cell compartment and remains heavily phosphorylated in the stalked cell compartment.

### The Model Agrees with the Phenotypes of Mutant Strains

The phenotypes of mutant cells provide crucial hints for deciphering the biochemical circuitry underlying any aspect of cell physiology. A mathematical model must be consistent with known phenotypes of relevant mutants. To make this test, we simulate cell cycle mutants of C. crescentus using exactly the same differential equations, parameter values, and initial conditions as for wild-type cells (Tables 1, 2, and 3), except for those modifications to parameters dictated by the nature of the mutation (Table 4). Our simulations of 16 classes of mutants are in agreement with experimentally observed phenotypes, as described here.

**Table 4:**
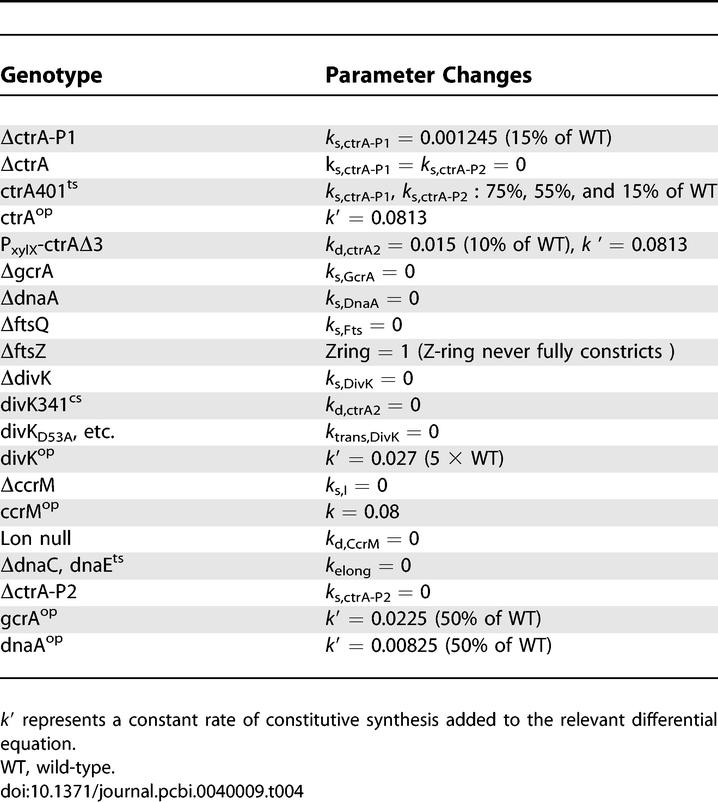
Altered Parameter Values for Mutant Simulations.

#### *ΔctrA-P1*.

This mutant is obtained by deleting the normal *ctrA* gene and placing a copy of *ctrA* near the terminus of the chromosome, where it is almost fully methylated throughout the cell cycle [40]. In this case, expression from the P1 promoter is greatly reduced, but expression from the P2 promoter is still possible [40]. As a result, a large proportion of mutated cells are longer than wild-type cells, and the appearance of the CtrA protein in the mutant cells is delayed relative to wild-type cells [40]. The mutant cells are able to divide, but with some time delay compared to wild-type cells. In our simulation (Figure S1), CtrA reappears with approximately 30 min delay, compared to wild-type cells. After that, the cell finishes the division cycle, as observed [40].

#### *ΔctrA*.

Experiments show that the CtrA depletion strain, *ΔctrA::spec + P_xylX_-ctrA* (the genomic copy of *ctrA* is disrupted, and a wild-type copy of the gene under control of a xylose-inducible promoter is integrated into the genome), is arrested and becomes filamentous without xylose inducer [46,47]. In our corresponding simulation (Figure S2), insufficient CtrA fails to stimulate expression of *ccrM* and *fts* genes. Therefore, the DNA remains hemimethylated, and the Z-ring cannot constrict. Cell division, but not metabolism and growth, is blocked, so the cell is expected to become filamentous.

#### *ctrA401^ts^*.

When the temperature-sensitive strain, *ctrA401^ts^*, is shifted to the restrictive temperature (37 °C), *ctrA* transcription is reduced by approximately 50%. This mutant has been studied thoroughly [15,29,30,32,36,47]. In our simulation (Figure S3), reduction of CtrA production to 70% of wild-type level leaves progression through the cell cycle unaffected. However, if *ctrA* expression lies between 70% and 35% of wild-type level, then cell division fails, but DNA synthesis continues repetitively, producing cells with multiple chromosomes. This curious phenotype was observed experimentally [30,36]. Lowering *ctrA* production rate (in our model) leads to decreasing expression (compared to wild-type) of *ccrM* and *fts* genes, with the latter becoming insufficient for Z-ring formation, as observed experimentally [29,32,41,47]. Since the Z-ring remains open, DivK∼P does not accumulate and CtrA stays elevated longer than in a wild-type cell, therefore lowering GcrA. The two latter effects result in DnaA being elevated so significantly that DNA replication is initiated in an undivided cell, despite an elevated level of CtrA and a depressed level of GcrA. When *ctrA* expression is reduced below 35% of wild-type level, the simulation is similar to *ΔctrA* (Figure S2).

#### *ctrA^op^* and *ctrAΔ3*.

A number of different mutations can cause increased levels of CtrA in cells: by constitutive expression of the gene, by producing a poorly degraded form of CtrA, by producing a constitutively active form of CtrA (not needing to be phosphorylated), or by combinations of these mutational strategies [30,38]. The phenotypes of these different mutant strains differ widely, from normal cell cycling to mixed arrest (in G1 and G2/M phases) to dominant G1 arrest. Although our model is consistent with some of these phenotypes, it is not consistent with them all because it does not take into account CtrA phosphorylation (see model assumptions in Materials and Methods).

Overproduction of CtrA does not interfere with normal cell cycling: the *ΔctrA1::spec + P_xylX_-ctrA* mutant grows and divides normally when CtrA is expressed constitutively from a xylose-inducible gene on a high copy-number plasmid [38]. By contrast, in our model (Figure S4), these cells arrest in G1 because they produce CtrA too early in the cell cycle. In reality, although G1 cells have an elevated level of CtrA, it is inactive (unphosphorylated), so these cells can proceed normally into S phase. This mutant emphasizes the importance of regulated CtrA phosphorylation. Since we do not yet account for CtrA activation and inactivation, this mutant is beyond the scope of our present model.

**Figure 6:**
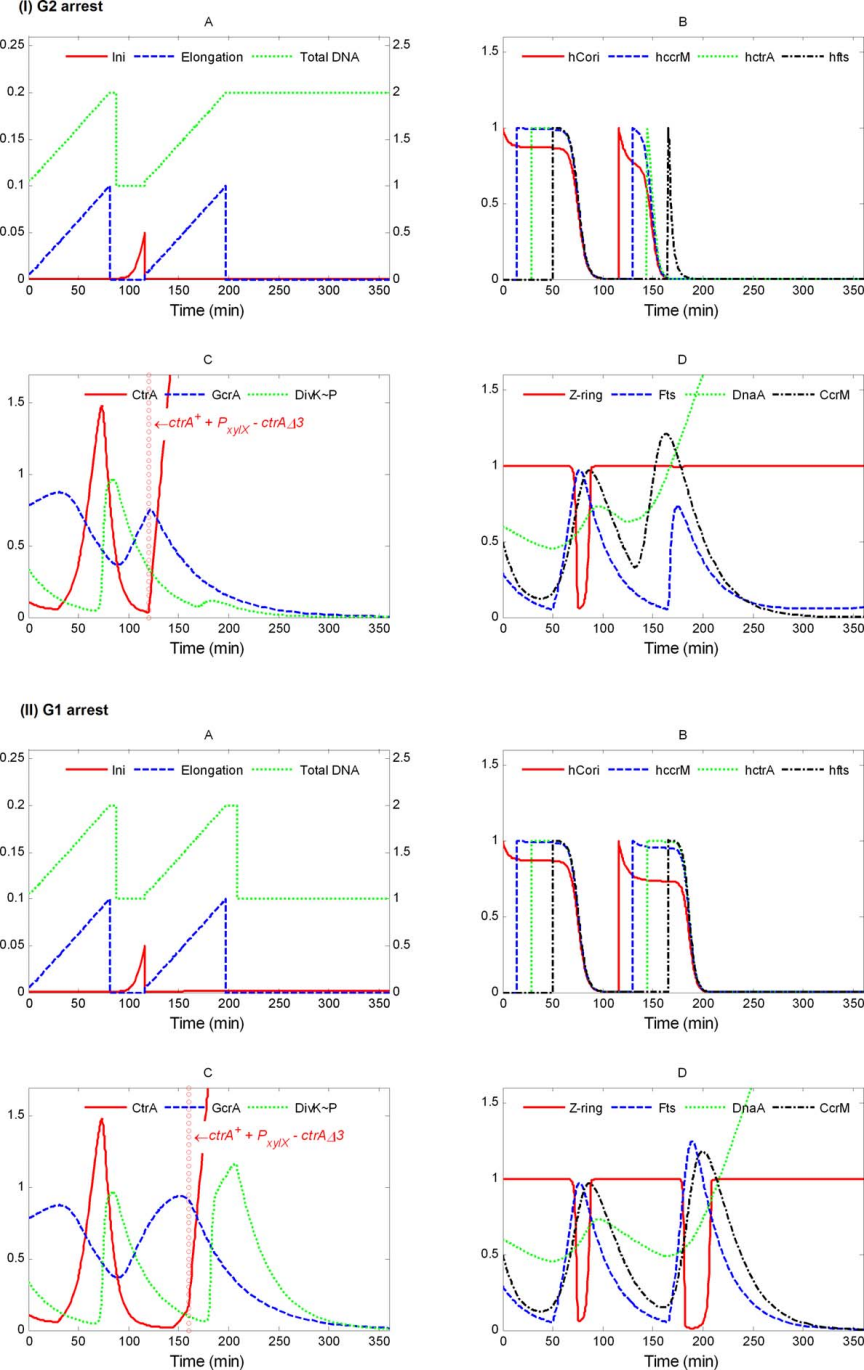
Simulation of *ctrA^+^ + P_xylX_ − ctrAΔ3* (Nondegradable CtrA) *k*′ = 0.0813 (constitutive synthesis rate), *k*_d,ctrA2_ = 0.015 (10% of WT). The vertical column of open circles here and on subsequent figures indicates the time at which the mutation is introduced.

When the genomic copy of *ctrA* is missing the coding sequence for the last three amino acids (*ctrAΔ3Ω*), the encoded mutant CtrA protein is more stable [38]. When this gene is introduced on a high copy-number plasmid (*ctrA^+^* [wild type] *+ P_xylX_-ctrAΔ3*) and the cells are grown on 0.2% xylose to overexpress the stable form of CtrA, then the mutant cells become filamentous, arresting either in G1 phase (unreplicated DNA) or in G2 phase (replicated DNA) [30]. Our model exhibits this behavior; whether cells arrest in G1 or G2 depends on cell cycle phase when CtrA production is induced (Figure 6). If CtrA production is induced in early-to-mid S phase, it does not interfere with DNA elongation, but does stimulate *ccrM* expression earlier than in wild-type cells, and the DNA becomes fully methylated promptly. Consequently, *fts* gene expression is extremely low, and cells become arrested with 2n chromosomes (Figure 6-I). On the other hand, if CtrA production is induced after *fts* genes have been turned on (Figure 6-II), then cell division proceeds on schedule, CtrA is not removed by proteolysis, and cells arrest in G1, unable to initiate a new round of DNA replication, because CtrA is blocking C_ori_.

#### ΔgcrA.

In the GcrA depletion strain, *ΔgcrA::Ω + P_xylX_-gcrA*, the chromosomal gene is disrupted and the *gcrA* coding sequence (under control of a xylose-inducible promoter) is integrated into the chromosome. In this mutant, when xylose is removed from the medium, the CtrA protein and *divK* transcription levels are decreased and *dnaA* expression is increased [15]. In our simulations (Figure S5), CtrA and DivK protein variations follow the experimentally observed trends after the first cycle. DnaA protein is elevated due to release from GcrA inhibition, as expected. In the simulation, GcrA depletion was introduced at *t* = 120 min, when DNA synthesis has already been successfully initiated. Since in our model progression of DNA replication is independent of GcrA, it persists until the end of the already initiated cycle. In real cells, GcrA is involved in maintenance of the replication machinery. Therefore, whether DNA replication will finish after the deletion of the *gcrA* gene depends on the abundance of GcrA protein relative to its rate of degradation. We are unaware of experimental studies relevant to this issue.

#### *ΔdnaA*.

When the *dnaA::Ω + P_xylX_::dnaA* mutant is shifted from xylose to glucose medium in order to block production of DnaA, DNA synthesis is arrested, and cell division is blocked [33,51]. Consequently, the expression of many genes is blocked. But when the cell is shifted from glucose back to xylose, DNA replication resumes, and the cell returns to a typical wild-type morphology. In our model mimicking this mutant, initiation of a new round of DNA synthesis fails due to insufficient DnaA protein (Figure S6). As a result, genes that are expressed only from hemimethylated DNA are never transcribed and the cell arrests in G1. In our simulation, the cell is viable when it is in the xylose medium, as *dnaA* is expressed constitutively (Figure S7, 120 < *t* < 300 min). If this simulated cell is shifted to glucose medium for a while (Figure S7, 300 < *t* < 370 min) and back to xylose medium again (*t* > 370 min), then it returns to normal cell cycling (Figure S7), in agreement with experimental observations [18].

#### ΔftsQ and ΔftsZ.

In the *ΔxylX-ftsQ::pBGPxQ* mutant, the chromosomal copy of *ftsQ* is deleted by recombination with a plasmid carrying the *ftsQ* gene under control of the P_xylX_ promoter. Blocking FtsQ production, by growing the cells on glucose, results in filamentous cells [61]. In our model, *ftsQ* is assumed to be essential for Z-ring formation and/or constriction. In the simulation (Figure S8), after *fts* expression is turned off, a cell can proceed through one round of DNA replication, but the Z-ring never fully constricts, and the cell never divides. Because growth continues (CtrA level is high), the cell is expected to become filamentous.

Similarly, when *ftsZ* is deleted (*ftsZ163ΔC + P_xylX_-ftsZ*), cytokinesis is inhibited, and cells become filamentous [67]. Our simulation (Figure S9), which assumes no Z-ring formation and constriction in the *ΔftsZ* mutant, produces results similar to those for *ΔftsQ* and is in agreement with observations.

#### ΔdivK.

**Figure 7:**
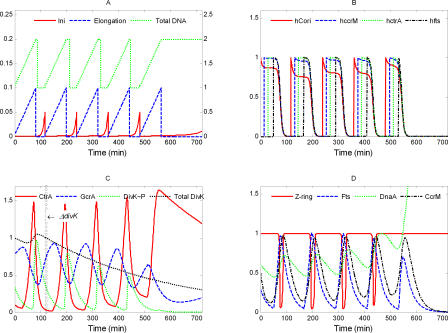
Simulation of *ΔdivK*: *k*_s,DivK_ = 0

Deletion of *divK* (*ΔdivK::Spec^R^*) was reported to be lethal [68,69], although how cells die when DivK is depleted has yet to be determined. In our simulation (Figure 7), after blocking expression of *divK*, cell divisions continue for some time, as divK protein is slowly lost from the cell (half-life = 350 min). Eventually, DNA replication and cell division stop with a high level of CtrA in the cell. In the terminal state, our simulated cell contains two DNA copies. The simulated cell eventually fails to divide because of insufficient levels of Fts proteins (Figure 7D). Compared to wild-type cells, the CtrA level is elevated, which stimulates production of Fts proteins and CcrM. The latter quickly methylates DNA, leaving a narrower window for expression of Fts proteins (Figure 7B). We were not able to find any experimental information about the DNA content of this mutant.

The model predicts that cells depleted of DivK may undergo four to five division cycles before dying, because (in the model) DivK protein is only slowly degraded, and its only job is to trigger degradation of CtrA. To the extent that other processes may render DivK nonfunctional and that DivK may have other essential roles in the cell [42,68], real cells may lose viability after DivK depletion more quickly than predicted here.

#### *divK341^cs^*.

**Figure 8:**
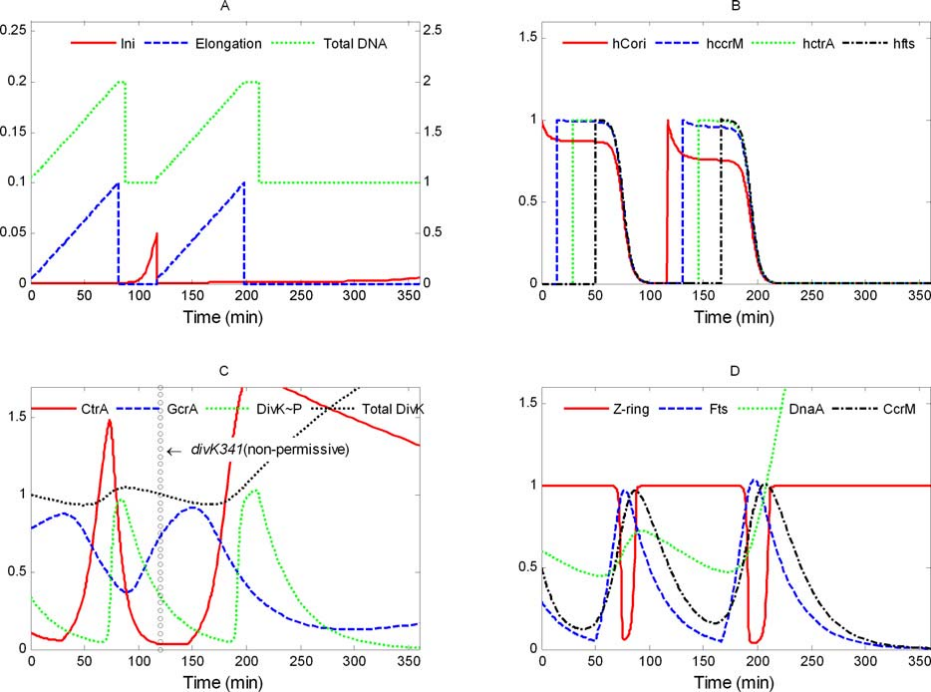
Simulation of *divK341^cs^*: *k*_d,ctrA2_ = 0

A point mutation (D90G) of *divK* creates a cold-sensitive allele, *divK341^cs^*, that (at the restrictive temperature, 25 °C) maintains a constant high level of CtrA and does not initiate DNA replication. Cells elongate, grow stalks, and become arrested in G1 phase with one chromosome [16,42,69]. In our simulation (Figure 8), we assume that, immediately after transfer to the restrictive temperature, DivK∼P loses its capacity to stimulate CtrA degradation [42]. After the origin of replication is initiated, DNA synthesis and methylation proceed normally in this case. The Z-ring fully constricts, but CtrA stays elevated. After the first division, DNA synthesis and cell division cease (in our simulation) because CtrA cannot be degraded. (The model also predicts a delayed initiation of DNA replication at *t* > 400 min, not evident in Figure 8. This is an artifact of our oversimplified differential equation for the DNA initiation variable, [Ini].)

If the mutation is introduced in our simulation before DNA replication starts, its start will be delayed because it takes longer for CtrA to be reduced (by the background degradation rate alone) to the replication-permissible level (Figure S10). Importantly, in the terminal state, the cell will block in G2 with replicated DNA.

If *divK341^cs^* is returned to the permissive temperature within about 70 min, cells recover to normal division cycles in our simulations (Figure S11; mutant is put into nonpermissive temperature at *t* = 120 min and returned to permissive temperature at *t* = 190 min), as in experiments [42].

#### *DivK_D53A_*, *DivK_E9A_*, and *DivK_D10A_.*

In this family of *DivK* amino acid replacement mutations, the phosphorylation of DivK is impaired, and DivK protein remains homogeneously dispersed through the cell [68]. In experiments, these mutant cells arrest in G1 phase and became filamentous [68]. These characteristics are observed in our simulations (Figure S12), which are similar to the simulation results (Figure 8) for the *divK341^cs^* mutant.

#### *divK^op^*.

**Figure 9:**
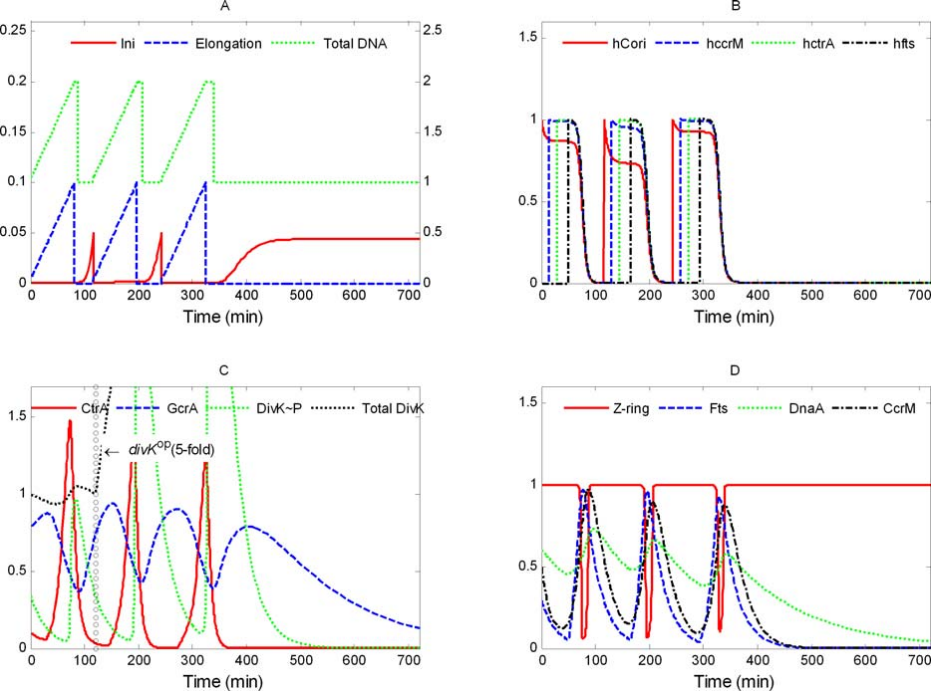
Simulation of *divK^op^*: *k*_s,DivK_ = 0, *k*′ = 0.027 (5 × WT)

In experiments, a *divK-cfp* fusion gene under control of the xylose-inducible promoter on a medium copy-number plasmid was introduced into wild-type cells [68]. When overexpressed, DivK was mislocalized, cell division was blocked, and cells became filamentous [68]. In our simulation, several-fold overeproduction of DivK causes arrest in G1 phase after one or more division cycles, depending on how much DivK is produced (Figure 9). Cells with lower *divK* overexpression undergo more cell cycles before arresting. These simulations are consistent with the observed phenotypes of *divK^op^* mutants. (Of course, mislocalization of DivK may have other effects on the cell cycle [68] that are not taken into account in our model.)

In [49], DivK was overexpressed from a high-copy plasmid. In our simulation, when DivK is strongly overexpressed (e.g., 20-fold), the cell becomes arrested in G2 phase. The lower level of CtrA is able to stimulate enough synthesis of DnaA to initiate a new round of DNA replication but fails to increase Fts sufficiently to trigger cell division. Thus the 20-fold over-expression mutant has a terminal phenotype similar to *ctrA^ts^* (Figure S3).

#### *ΔccrM*.

**Figure 10:**
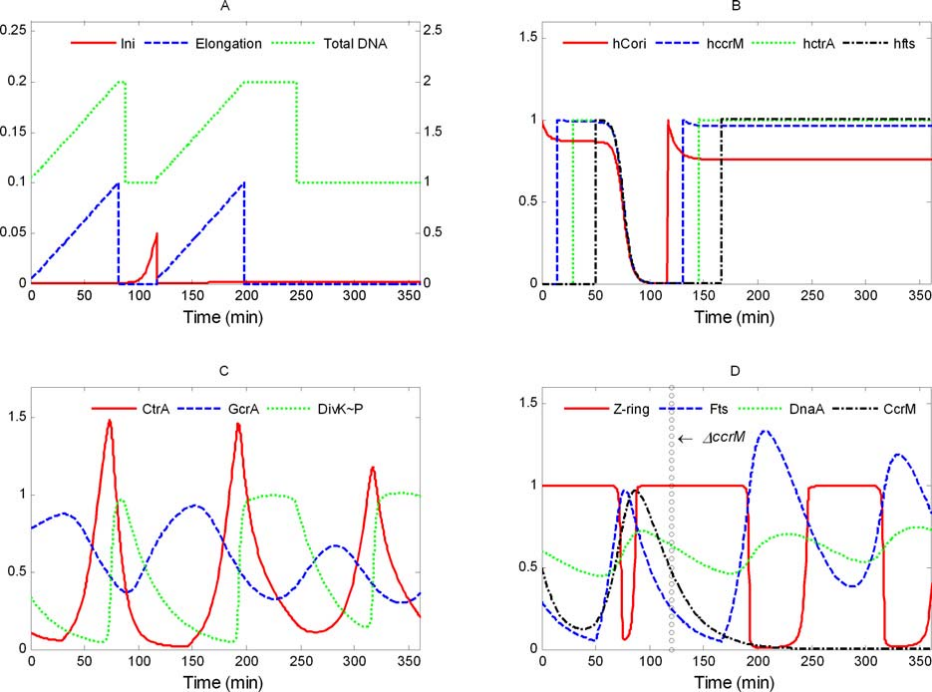
Simulation of *ΔccrM*: *k*_s,I_ = 0

In a CcrM-depleted strain (Δ*ccrM* + *P_xylX_-ccrM*, where the chromosomal *ccrM* locus is inactivated and CcrM is supplied from a xylose-inducible promoter on a low copy-number plasmid), methylation of the new DNA strand ceases, when cells are grown on glucose [39,40]. Cell growth and DNA replication cease in 6–8 h. Cell viability starts dropping after approximately 4 h. Our simulation of this mutant (Figure 10) shows that, without sufficient CcrM, DNA remains hemimethylated; hence, initiation of a new round of DNA replication is repressed. In addition, *ctrA* and *fts* genes can now be periodically expressed in the absence of DNA replication, which is usually required to return these genetic loci to a hemimethylated state. Elevated, periodic variations of Fts proteins can induce premature constriction of the Z-ring, if other relevant conditions are also satisfied. That leads to cell division without DNA replication, suggesting a precipitous loss of viability of these mutant cells, as observed experimentally [39,40].

#### *ccrM^op^*.

**Figure 11:**
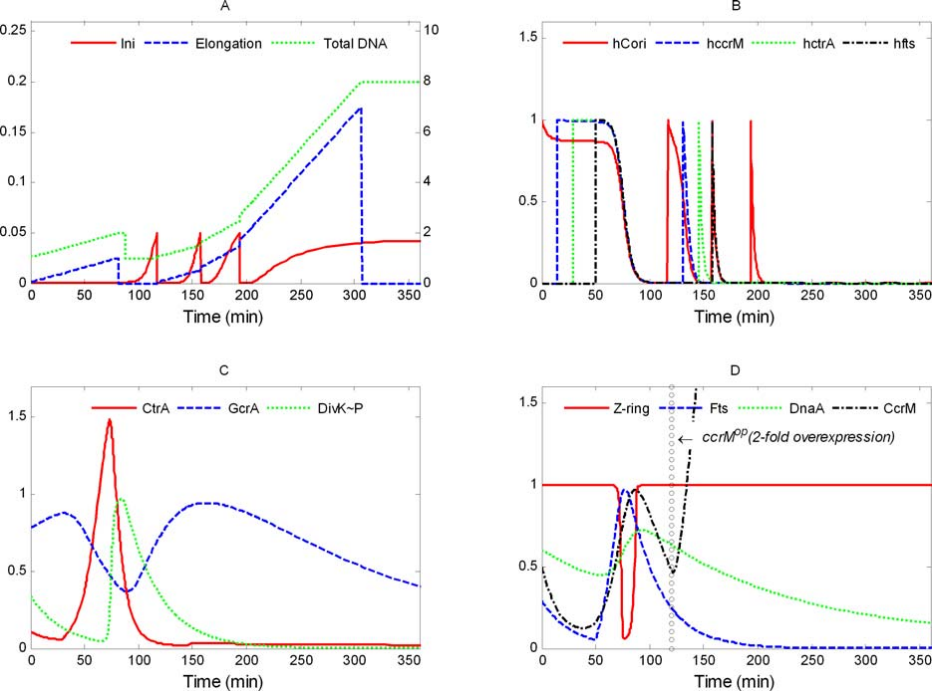
Simulation of *ccrM^op^*: *k*′ = 0.16 (2 × WT)

In the CcrM overproduction mutant, the wild-type *ccrM* gene is supplemented by a second chromosomal copy expressed constitutively from a P_lac_ promoter. CcrM overproduction causes rapid methylation of newly synthesized strands of DNA in experiments; some cells accumulate multiple chromosomes because additional initiations of DNA synthesis occur before cell separation [52]. About half of the mutant cells are longer than wild-type, and cell division is morphologically aberrant [52]. In our simulation (Figure 11), a constitutively high level of CcrM accelerates methylation of newly synthesized DNA; hence, *ctrA* has little chance to be transcribed, and consequently, the Z-ring never fully constricts. At the same time, conditions are right for repeated rounds of DNA synthesis. The number of excess chromosomes per cell depends on the extent of overexpression of *ccrM* in our simulations, as can be seen by comparing Figure 11 (2-fold overexpression) with Figure S13 (50% overexpression).

#### Lon null mutant.

Lon protease is required for CcrM degradation in living cells [58]. Without it, CcrM is present at high levels throughout the cell cycle, similar to the *ccrM^op^* mutation. As a result, cells become filamentous with multiple chromosomes [58]. Our simulation (Figure S14) is consistent with the observed phenotype.

#### *ΔdnaC* and *dnaE^ts^.*

Elongation of DNA during S phase ceases in cells depleted of DnaC [70]. The *dnaE^ts^* mutant also blocks DNA elongation at the nonpermissive temperature (37 °C), and cells arrest with an undetectable level of CtrA [41]. To simulate these mutants, DNA elongation was interrupted at different times during the cell cycle (Figure S15). If DNA elongation is blocked in early-to-mid S phase, then few *fts* genes can be successfully expressed, and the Z-ring stays unconstricted. The mutant cell arrests in an early S phase (GcrA high, CtrA low). If the knock-out is made late in S phase, then cell division occurs normally, provided that incompletely replicated chromosomes can be separated to progeny cells. The progeny cells would then arrest pre-S phase. In reality, incompletely replicated chromosomes may prevent cell division [71], in which case, cells would arrest in a late predivisional stage. We do not include an unreplicated-DNA checkpoint in our model.

### The Model Predicts Phenotypes of Novel Mutants

#### ΔctrA-P2.

**Figure 12:**
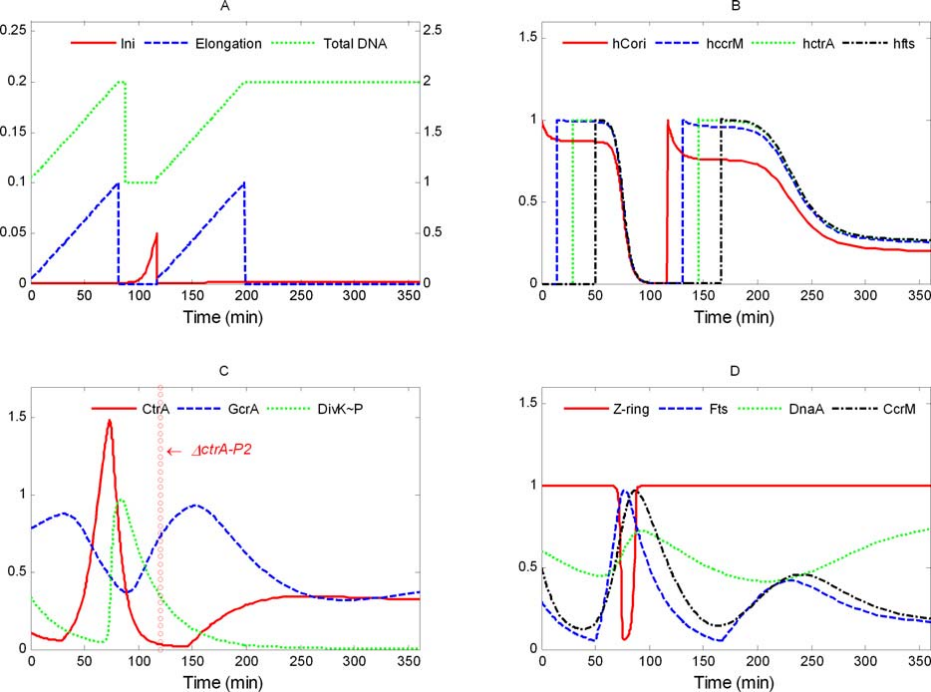
Simulation of *ΔctrA-P2*: *k*_s,CtrA-P2_ = 0

Based on our simulations (Figure 12), the cell cycle in a *ctrA-P2* deletion mutant should arrest in G2 (replicated chromosome) as long (filamentous) cells. CtrA never reaches a high concentration and hence fails to activate much expression of *ccrM* and *fts* genes*.* Without sufficient Fts proteins, the Z-ring does not constrict. At the same time, DNA replication can continue, once it has been initiated. However, new DNA strands cannot be fully methylated because of insufficient CcrM, and thus a new round of DNA replication cannot be initiated.

#### *gcrA^op^*.

**Figure 13:**
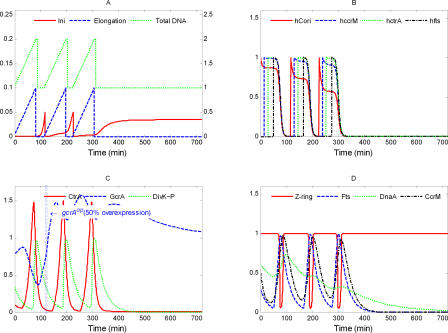
Simulation of *gcrA^op^*: *k*′ = 0.0225 (50% of WT)

No mutant overproducing GcrA has been reported in the literature. Our model predicts that small overexpression of *gcrA* (∼110% in our simulations) speeds up production of CtrA, which accelerates progression through the cell cycle (Figure S16). Higher levels of GcrA (150%–200% overexpression in our simulations) cause a significant decrease of DnaA protein. As DnaA level drops, eventually DNA synthesis cannot be initiated, and the cell should arrest in G1 phase (Figure 13).

#### dnaA^op^.

**Figure 14:**
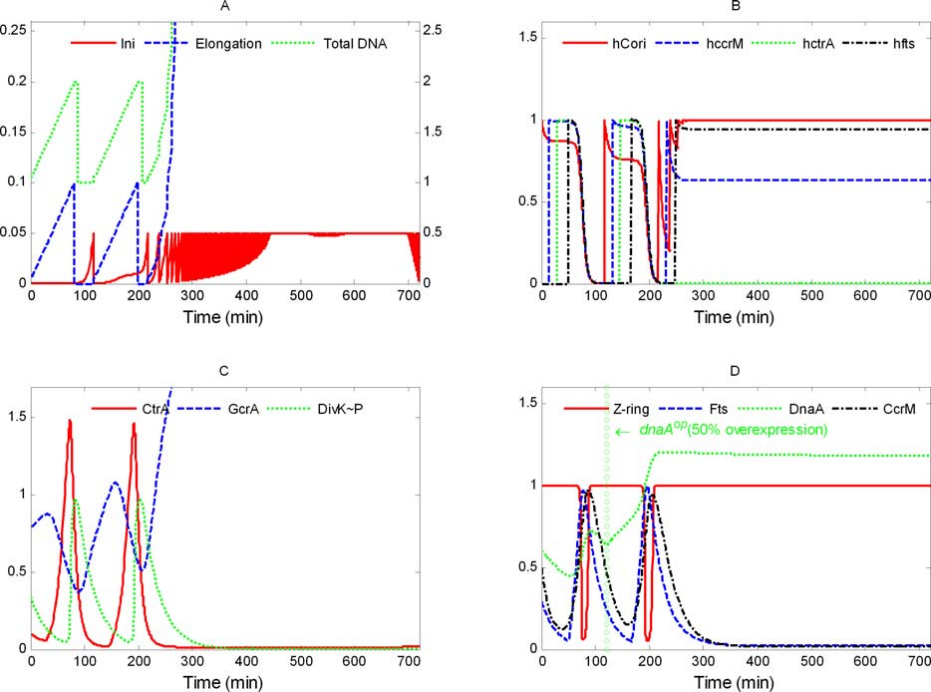
Simulation of *dnaA^op^*: *k*′′ = 0.00825 (50% of WT)

Phenotypes of *dnaA* overexpressing mutants have not been reported in the literature, to our knowledge. According to our simulations, modest overexpression of *dnaA* (110%) stimulates *gcrA* expression quicker than in wild-type cells. High levels of DnaA and GcrA, combined with low CtrA, accelerate initiation of DNA replication and speed up the cell cycle somewhat (∼100 min in our simulation; Figure S17). If *dnaA* is overexpressed by 150% or more, the elevated level of DnaA protein causes rapid, repeated initiations of DNA replication forks, in our simulations (Figure 14), suggesting that at least several initiations of DNA replication may take place. Our model permits instantaneous initiation of DNA replication as soon as the initiation condition is satisfied, whereas in real cells, re-initiation must take some minimal time. Hence, our prediction is that several re-initiations of DNA replication may take place in *dnaA^op^* cells without further cell divisions.

## Discussion

We propose (Figure 3) a realistic mechanism for regulating the cell division cycle of stalked cells of C. crescentus. The mechanism includes three master-regulatory proteins (GcrA, DnaA, and CtrA), a DNA methylase (CcrM), Z-ring components (Fts proteins), and an end-of-cycle protein (DivK) in its inactive and active (phosphorylated) forms. Cytokinesis is represented by a phenomenological variable that describes the extent of constriction of the Z-ring. DNA synthesis is described in terms of initiation, elongation, and termination. We assume that initiation of DNA replication requires high DnaA and GcrA, low CtrA, and full methylation of the origin site, and that the rate of DNA elongation is independent of DnaA, GcrA, and CtrA, and is almost linear. Transcription of some genes occurs only from an unmethylated DNA sequence; hence, the expression of such genes depends on their location on the newly synthesized DNA strand. Compartmentation in the predivisional cell is assumed to result in localization of phosphorylated DivK to the stalked compartment of the dividing cell, promoting CtrA degradation there.

These assumptions are formulated as a mathematical model (Table 1) consisting of 16 nonlinear, ordinary differential equations for seven proteins, the state of the Z-ring, the progression of DNA synthesis, and the methylation state of five gene sites on the DNA. The rate equations entail 44 parameters (rate constants, binding constants, and thresholds; Table 2) that need to be determined by fitting the model to specific experimental observations. For the present, parameter estimation is done by trial and error, so we can only claim that our model equations and parameter set are sufficient to account for many properties of cell cycle control in C. crescentus. Because we fit the model to many different mutant phenotypes, we have a wealth of data to fix the parameters and to provide meaningful confirmation of the mechanism. Table 2 is in no sense an optimal parameter set, nor can we quantify how robust the system is, although our experience suggests that the model is quite hardy.

Our present model is based heavily on an earlier conjecture [17] that the C. crescentus cell cycle is controlled by a bistable switch, created by positive feedback in the molecular circuitry of the *ctrA* gene. In that conjecture, the switch is flipped from the off-state (CtrA low) to the on-state (CtrA high) by GcrA accumulation as cells enter S phase, and then switched back to the off-state by DivK activation (phosphorylation) as cells divide (the CtrA–DivK negative feedback loop). The original model did not account for the ways in which gene expression is linked to DNA methylation, thereby anchoring the protein interaction network to the progression of DNA replication forks. By incorporating DNA synthesis and methylation into the Brazhnik–Tyson model, the present model provides a more satisfactory account of cell cycle regulation in C. crescentus, and it can be tested by comparison to a broad spectrum of mutant phenotypes. Because the new model successfully reproduces the behavior of wild-type and mutant cells in many quantitative details, we conclude that our present understanding of the control system (Figure 3 and Table 1), properly interpreted, is accurate and adequate. On the other hand, the proposed mechanism must be considered as an evolving hypothesis that will be continually examined, revised, and improved as new observations tell us more about the control system. Some obvious improvements to the model include refined criteria for DNA initiation, regulated phosphorylation of CtrA, spatial localization of proteins, inclusion of a swarmer cell compartment, and an account of the swarmer-to-stalk cell transition.

Finally, most of division-control proteins (such as CtrA, DivK, CcrM, FtsZ, and FtsQ) are conserved among α-proteobacteria [72], suggesting that the computational model proposed here for C. crescentus may prove applicable to other types of α-proteobacteria, including symbiotic nitrogen-fixing genera (*Rhizobia*) and pathogenic genera (Brucella spp., *Coxiella* spp, etc.)

## Materials and Methods

### Scope of the model.

To understand the molecular logic of cell cycle regulation in C. crescentus, we constructed a mathematical model of the temporal dynamics of the regulatory genes and proteins. Following standard rules of chemical kinetics, we converted the wiring diagram in Figure 3 into a set of rate equations describing the temporal dynamics of the model. Justification of our approach is described in detail in [17].

Our model includes:

Seven proteins: DnaA, GcrA, CtrA, CcrM, DivK (inactive), and DivK∼P (phosphorylated, active form), and a “representative” Fts protein.

Two phenomenological variables, *Z* (the state of closure of the septal Z-ring) and *I* (introducing a delay between activation of *ccrM* transcription and later activation of CcrM protein production).

The progression of DNA replication (including initiation, elongation, and termination) and its methylation (including probabilities of hemimethylation of *ccrM*, *ctrA*, *dnaA*, and *fts* genes, and of the replication origin site, C_ori_).

Accordingly, our mathematical model consists of 16 nonlinear differential equations presented in Table 1, including 28 kinetic constants (*k*'s), 11 binding constants (*J*'s), and five thresholds (*θ*'s). Our choice of parameter values is given in Table 2.

### Assumptions of the model.

A common trend in developing complex models in molecular cell biology is to start from a simple coarse-grained (“phenomenological”) model and then refine and expand it step by step (as data become available) into an increasingly more comprehensive model. (A good example is the progression of models of the budding yeast cell cycle [2,4,73].) We have taken this approach in our study of the C. crescentus cell cycle. We have limited the scope of our model so that it can be based largely on experimental observations, is not overwhelmed with assumptions, and is able to make predictions. Obviously, at any stage of modeling there will be facts that have not yet been incorporated and thus are out of the scope of the model. Our modeling assumptions are described here.

First, we propose to model, at this stage, only the average behavior of cells and do not address naturally occurring fluctuations in cell cycle progression.

Second, the rise of DivK∼P in stalked compartments after constriction of the Z-ring is a necessary, but not sufficient, condition for CtrA degradation. In our coarse-grained model of CtrA proteolysis, we use DivK∼P as a signal for starting rapid degradation of CtrA. In other words, DivK∼P determines when the degradation of CtrA is turned on, but the how (the machinery that degrades CtrA, involving RcdA, CpdR, and ClpXP) is assumed to be there when needed and is not modeled at present.

Third, CtrA is activated by phosphorylation (by kinases CckA and DivL), and a complete model of the *Caulobacter* cell cycle should take this into account. Unfortunately, little is known about the phosphorylation and dephosphorylation of CtrA and how these processes are temporally regulated. During the division cycle of wild-type cells, the levels of CtrA and CtrA∼P rise and fall together [22,46], so we need not distinguish between the two forms. Therefore, in the current model, we keep track of CtrA synthesis and degradation only, assuming that CtrA∼P is a fixed fraction of total CtrA. This assumption, though a great oversimplification, is harmless enough for most of the mutants we consider in this paper. But it seems to cause serious problems for exactly those mutants (*ctrA^op^*, *ctrAΔ3*, *ctrAD51E*, and *ctrAD51EΔ3* in wild-type background) that interfere with normal synthesis, degradation, or activation of CtrA [34]. Later versions of the model will have to include CtrA∼P as a variable, when we have a better of idea of the mechanisms controlling CtrA phosphorylation.

It is known that DivK∼P promotes the proteolysis of CtrA∼P [42] and negatively regulates CckA activity, thereby reducing phosphorylation of CtrA [49,74]. Hence, DivK∼P works to eliminate CtrA∼P activity by two independent pathways. We lump these two effects together as a single DivK∼P promoted reaction for removing active CtrA.

Fourth, the *dnaA* locus is very close to the origin site (C_ori_) [28]. Within its promoter, potential CtrA and DnaA boxes and methylation sites exist for regulating its expression [20,34,52,55]. GcrA is a repressor for *dnaA* expression [15], and CtrA seems to be an activator [32]. However, DnaA protein concentration varies very little during the *Caulobacter* cell cycle [55]. Although we include the regulatory signals in the model, they do not much affect the dynamics of a stalked cell because DnaA level is nearly constant throughout the cell cycle due to DnaA's long half-life.

Fifth, initiation of DNA replication is triggered by the combined conditions of low CtrA, high DnaA, and fully methylated DNA origin site. In addition, initiation requires sufficient replication machinery, which is correlated to a high level of GcrA. We combine these regulatory effects into a single term. We assume that once initiation of DNA replication is successful, DNA elongation starts immediately. Elongation of new DNA strands is linear in time until it finishes, based on experimental data indicating that the speed of DNA replication in C. crescentus is almost constant [54].

Sixth, full constriction of the Z-ring requires accumulation and activation of a number of proteins, including FtsZ, FtsQ, FtsA, and FtsW, some of which are stimulated by CtrA. To simplify the model, we use Fts as a combined component to relay the signal from CtrA to Z-ring constriction. The transition from Z-ring open (= 1) to fully constricted (= 0) is modeled as a Goldbeter-Koshland ultrasensitive switch [75].

Seventh, we include the effects of DNA methylation on gene expression in our model because these effects mediate important feedback loops between DNA synthesis and the master regulatory proteins, and because DNA methylation can be a useful target for new drug development. In our model, the genes *ccrM*, *dnaA*, *ctrA*, and *fts* as well as the origin of DNA replication are regulated by methylation.

Methylation plays a minor role in the regulation of GcrA production [19], so we disregard it in our model. We allow a modest contribution of DNA methylation to regulating the production of DnaA. *ccrM* gene expression is significantly affected by its methylation state [40,57]. The activity of *ctrA-P1* is known to depend on hemimethylation [36], and the activity of *ctrA-P2* seems to depend in some other way on DNA replication [37]. For simplicity, we assume that both *ctrA* promoters are turned on by hemimethylation of the gene.

Among *fts* genes, the *ftsZ* promoter has a methylation site [40,53], but the *ftsQ* promoter does not [41]. Scanning the *ftsQ* gene for the consensus sequence GANTC using the Regulatory Sequence Analysis Tools (http://rsat.ulb.ac.be/rsat/), we found a GAGTC segment in the coding sequence, suggesting that the *ftsQ* gene might also be affected by methylation. Since our “Fts” variable is a combination of Fts proteins, we conclude that our *fts* gene should be regulated by methylation.

The effects of methylation on gene promoters and C_ori_ are described by probabilities to be methylated or hemimethylated during the cell cycle. The probabilities (*h*_.._ variables) are in turn controlled by the progression of DNA replication and by the activity of CcrM [52,53].

Eighth, *ccrM* transcription is tightly regulated by CtrA protein, but accumulation of CcrM protein shows a noticeable delay from the transcriptional activation of its gene [37], resulting in delayed activation of DNA methylation [57]. This delay is mimicked in our model by an intermediate variable *I* in the CtrA-to-CcrM pathway.

Ninth, we recognize the importance of spatial controls in the *Caulobacter* cell cycle. However, at this stage, we are trying to model the stalked cell cycle as far as possible without explicitly tracking the spatial localization of regulatory proteins. That would require a more sophisticated mathematical framework and is planned for the next stage of the model. As the result of this simplification, our model makes no distinction between the stalked and swarmer parts of the predivisional cell. Right after compartmentation and before cytokinesis, we keep track of proteins in the stalked cell compartment only. At this stage, the distinction between swarmer and stalked cells is made by the phosphorylation state of DivK (being completely phosphorylated in the stalked compartment).

Tenth, we assume cells grow steadily in time, with a mass-doubling time of about 120 min and with the accumulated material shed at each division in the swarmer cell. In the present model, there is no coupling between cell growth and division, as in our models of eukaryotic cell proliferation [10]. Hence, there is no need for us to keep track of cell size, except to notice that if cell division is delayed or blocked, then the stalked cell will grow longer than normal and eventually be described as having a filamentous morphology.

### Parameter values and initial conditions.

Parameter values for our model (Table 2) were determined from available experimental data, wherever possible. Rate constants of degradation were estimated from experimentally observed half-lives of proteins. Rate constants of protein synthesis were adjusted to fit variations of protein concentration observed in experiments. Parameter values of Z-ring dynamics were set to be consistent with observed durations of the open (∼100 min) and constricted (∼20 min) states of the Z-ring [59]. Rate constants of DivK phosphorylation and dephosphorylation were estimated from the difference of DivK∼P concentration before and after Z-ring closing in predivisional cells [63]. Successful initiation of DNA replication depends on satisfying four requirements: low CtrA ([CtrA] < θ_CtrA_), high DnaA ([DnaA] > θ_DnaA_), high GcrA ([GcrA] > θ_GcrA_), and a fully methylated origin site (*h*_cori_ < θ_Cori_). The thresholds were adjusted to position the onset of the S phase correctly in wild-type cells. Replication-fork progression (elongation) begins at each successful initiation ([Ini] = 0.05) and stops when DNA replication is complete ([Elong] = 1).The constant rate of elongation is consistent with an 80-min delay for copying the chromosome. Due to the constant rate of DNA replication, those genes that must be hemimethylated in order to be transcribed will be expressed in a temporal sequence determined by their positions on the chromosome from the origin of replication [39,76]. To model this effect, the variable *h_gene_* is set to 1 (hemimethylated) when [Elong] = distance of *gene* from C_ori_. Some time thereafter, when CcrM activity is high, the *h_gene_* decays exponentially back to 0 (fully methylated). Most Hill function exponents are assumed to be 2, with a higher value (*n*_H_ = 4) where sharper switching was required. Initial conditions (Table 3) were taken to represent the beginning of a stalked cell cycle in a wild-type cell.

### Simulation of mutants.

The phenotypes of relevant mutants were collected from the literature. To simulate each mutant, we use exactly the same equations (Table 1) and parameter values (Table 2) except for values of those parameters directly affected by the mutation (Table 4). Mutations are introduced in our model after 120 min of simulation of the wild-type cell. For gene deletion, the rate of synthesis of the corresponding protein is set to zero. For gene overexpression, an additional constant rate of synthesis of the corresponding protein is introduced into the equations, because proteins are typically overexpressed from an extra copy of the gene under control of an inducible promoter. For heat- or cold-sensitive mutants, the relevant rate constant(s) retains its wild-type value at the permissive temperature and is set to zero at the restrictive temperature. For partial deletions, the relevant parameter value is assumed to lie between 0% and 100% of the wild-type value, according to the experimental characterization of the mutation.

Equations of the model were solved numerically with Matlab 2006a (The MathWorks). Machine-readable files for reproducing our simulations are made available in Text S1 and on our Web site (http://mpf.biol.vt.edu/research/caulobacter/pp/).

## Supporting Information

Figure S1 Simulation of *ΔctrA-P1*: *k*_s,ctrA-P1_ = 0.001245 (15% of WT) The vertical column of open circles here and on subsequent figures indicates the time at which the mutation is introduced. WT, wild-type.
(573 KB TIF)

Figure S2 Simulation of *ΔctrA*: *k*_s,ctrA-P1_ = *k*_s,ctrA-P2_ = 0 (550 KB TIF)

Figure S3 Simulation of *ctrA401^ts^*
(I) *k*_s,ctrA-P1_ = 0.006225, *k*_s,ctrA-P2_ = 0.05475.
(II) *k*_s,ctrA-P1_ = 0.004565, *k*_s,ctrA-P2_ = 0.04015.
(III) *k*_s,ctrA-P1_ = 0.00057, *k*_s,ctrA-P2_ = 0.01095
(1.7 MB TIF)

Figure S4 Simulation of *ctrA^op^*: Add Constitutive Synthesis at Rate *k* ^′^ = 0.0813 (557 KB TIF)

Figure S5 Simulation of *ΔgcrA*: *k*_s,GcrA_ = 0 (563 KB TIF)

Figure S6 Simulation of *ΔdnaA*: *k*_s,DnaA_ = 0 (564 KB TIF)

Figure S7 Simulation of Rescue of *ΔdnaA*
*k*_s,DnaA_ = 0.0165 (WT) for 0 < *t* < 120 min, *k*_s,DnaA_ = 0 and *k* ^′^ = 0.042 (constitutive expression from xylose-inducer promoter) for 120 < *t* < 300 min, *k*_s,DnaA_ = 0 and *k* ^′^ = 0 for 300 < *t* < 370 min, and *k*_s,DnaA_ = 0 and *k* ^′^ = 0.042 for *t* > 370 min.
(623 KB TIF)

Figure S8 Simulation of *ΔftsQ*: *k*_s,Fts_ = 0 (558 KB TIF)

Figure S9 Simulation of *ΔftsZ*: Zring = 1 (560 KB TIF)

Figure S10 Simulation of *divK341^cs^*: *k*_d,ctrA2_ = 0 at *t* = 80 min (561 KB TIF)

Figure S11 Simulation of Rescue of *divK341^cs^*
Shift to restrictive temperature (*k*_d,ctrA2_ = 0) at *t* = 120 min and back to permissive temperature (*k*_d,ctrA2_ = 0.15) at *t* = 190 min.
(649 KB TIF)

Figure S12 Simulation of Nonphosphorylatable DivK Mutants: *k*_trans,DivK_ = 0 (570 KB TIF)

Figure S13 Simulation of *ccrM^op^*: *k* ^′^ = 0.04 (50% of WT) WT, wild-type.
(569 KB TIF)

Figure S14 Simulation of the *Lon Null* Mutant: *k*_d,CcrM_ = 0 (Lon Protease Degrades CcrM) (555 KB TIF)

Figure S15 Simulation of *dnaC303* and *dnaE^ts^* Mutants: *k*_elong_ = 0 Mutation expressed at different times, as indicated.
(1.6 MB TIF)

Figure S16 Simulation of *gcrA^op^*: *k* ^′^ = 0.0045 (10% of WT) WT, wild-type.
(639 KB TIF)

Figure S17 Simulation of *dnaA^op^*: *k* ^′^ = 0.00165 (10% of WT) WT, wild-type.
(649 KB TIF)

Text S1 Supplementary Codes for Simulation (65 KB DOC)

Text S2 Genes and Proteins That Appear in This Paper (46 KB DOC)
