# Supplementary figures and images for: A Quantitative Study of the Division Cycle of Caulobacter crescentus Stalked Cells
Source: PLoS Comput Biol. 2008 Jan 25;4(1):e9. doi: 10.1371/journal.pcbi.0040009 (PMC2217572; doi:10.1371/journal.pcbi.0040009)

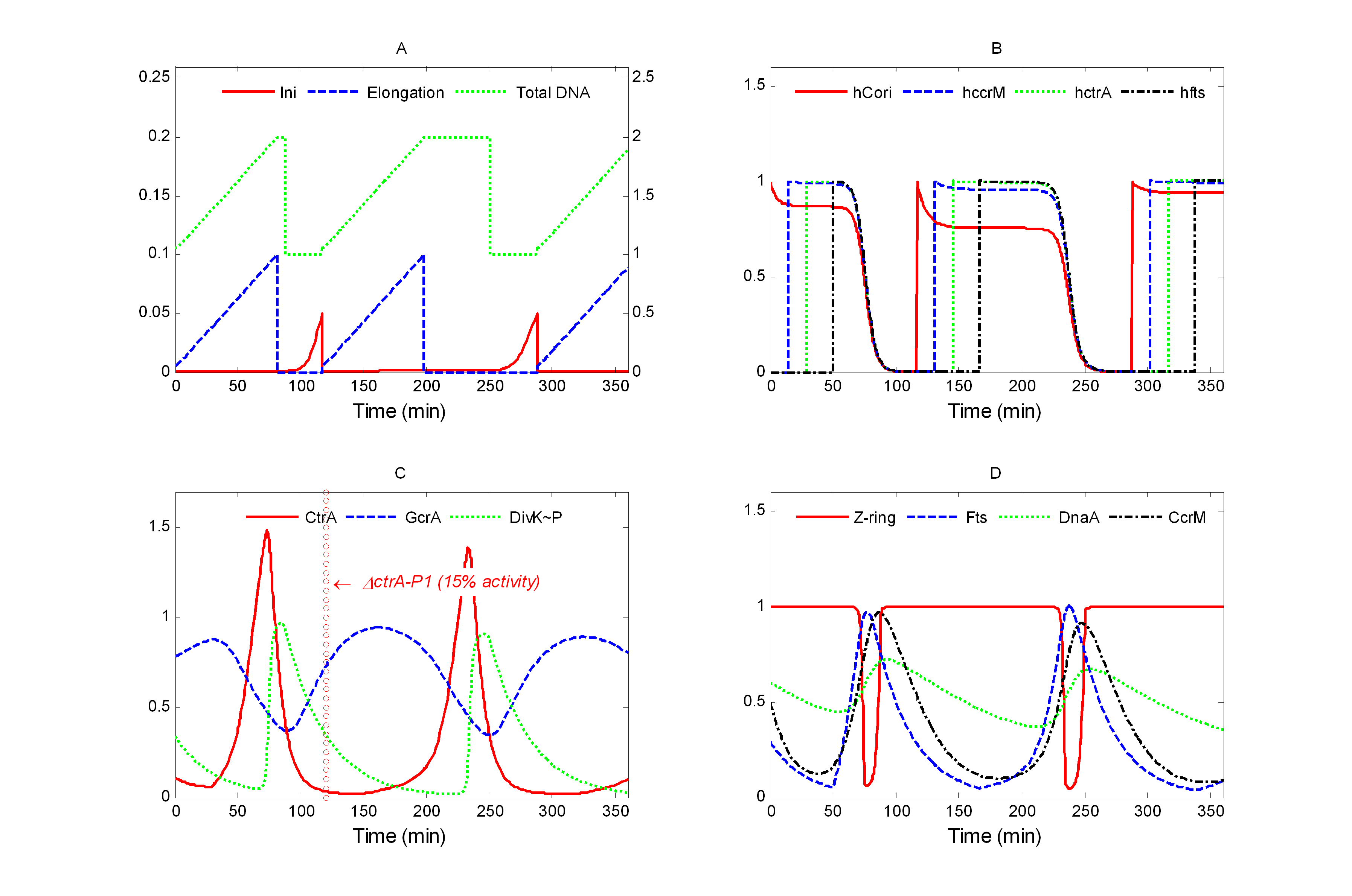

Supplement: Figure S1 — The vertical column of open circles here and on subsequent figures indicates the time at which the mutation is introduced. WT, wild-type. (573 KB TIF) [file pcbi.0040009.sg001.tif]

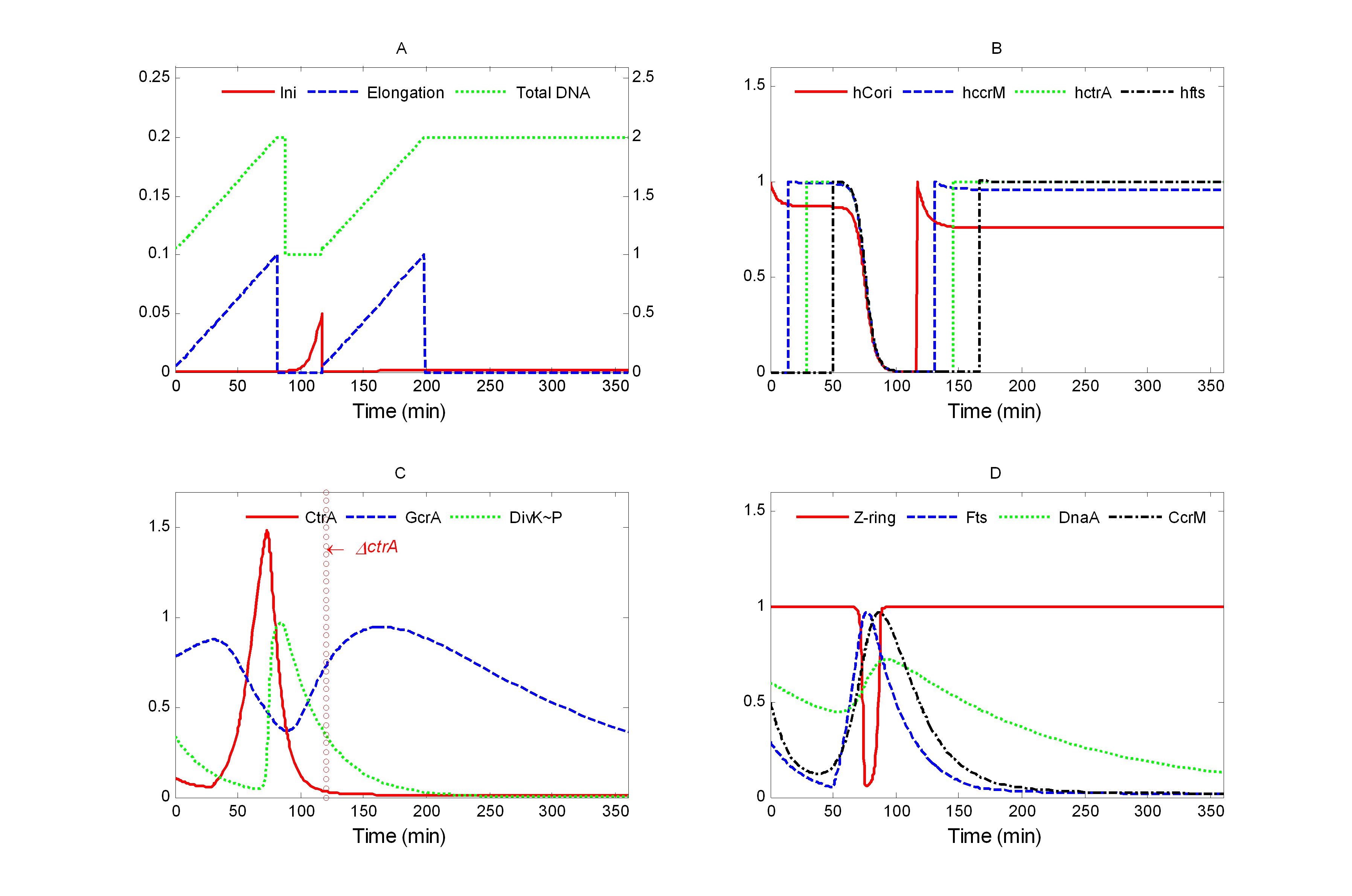

Supplement: Figure S2 — (550 KB TIF) [file pcbi.0040009.sg002.tif]

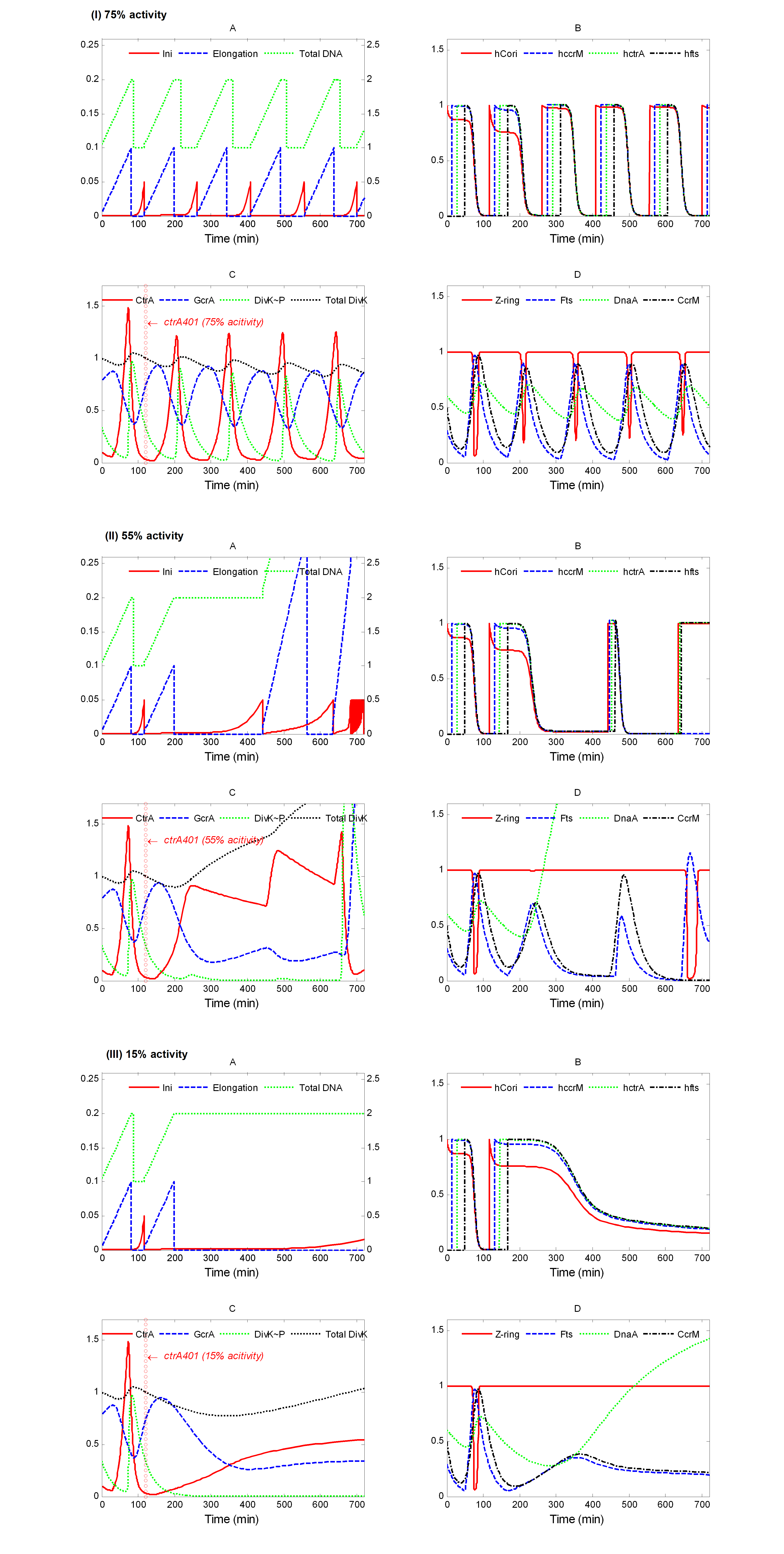

Supplement: Figure S3 — (I) k s,ctrA-P1 = 0.006225, k s,ctrA-P2 = 0.05475. (II) k s,ctrA-P1 = 0.004565, k s,ctrA-P2 = 0.04015. (III) k s,ctrA-P1 = 0.00057, k s,ctrA-P2 = 0.01095 (1.7 MB TIF) [file pcbi.0040009.sg003.tif]

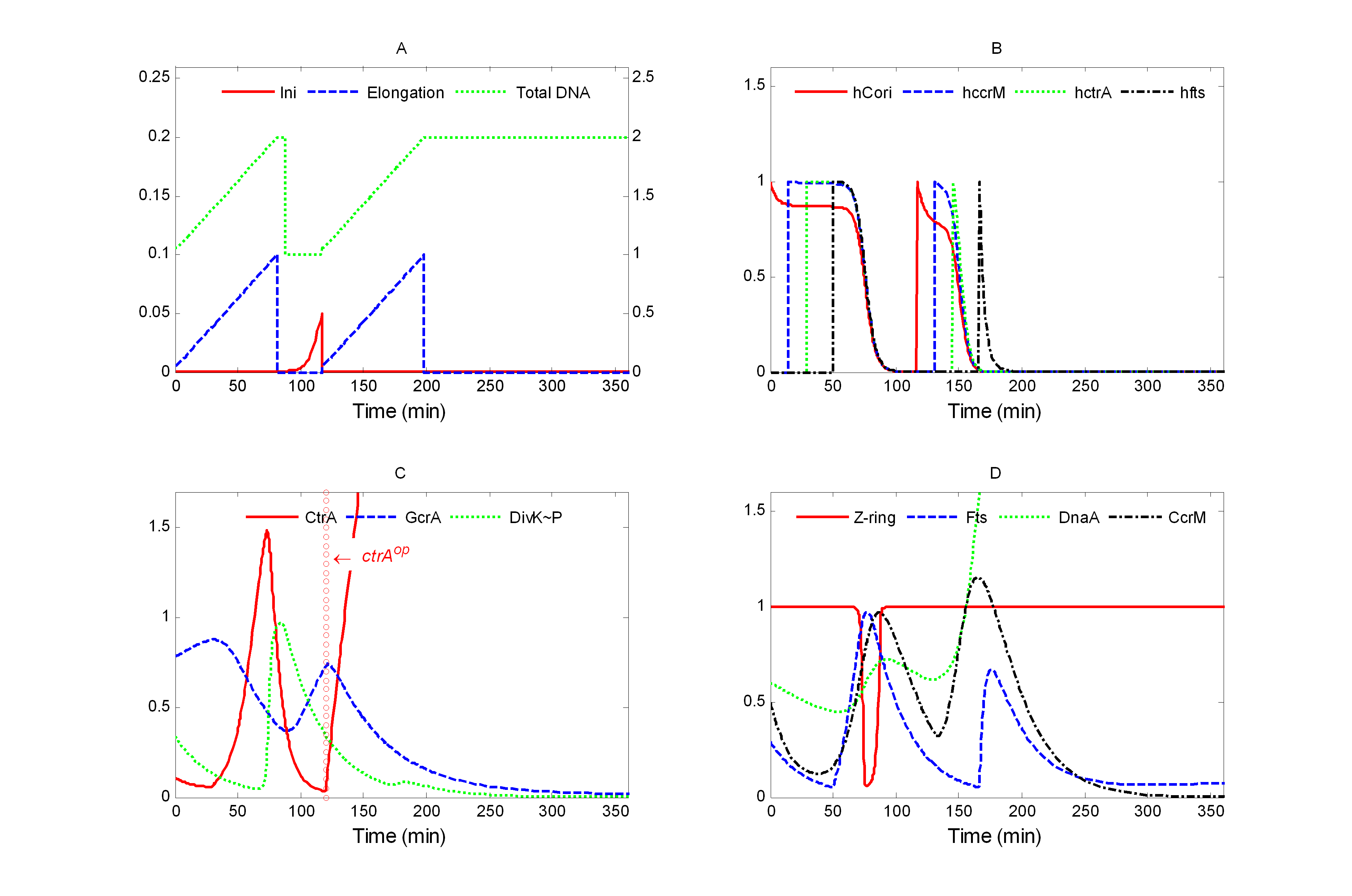

Supplement: Figure S4 — (557 KB TIF) [file pcbi.0040009.sg004.tif]

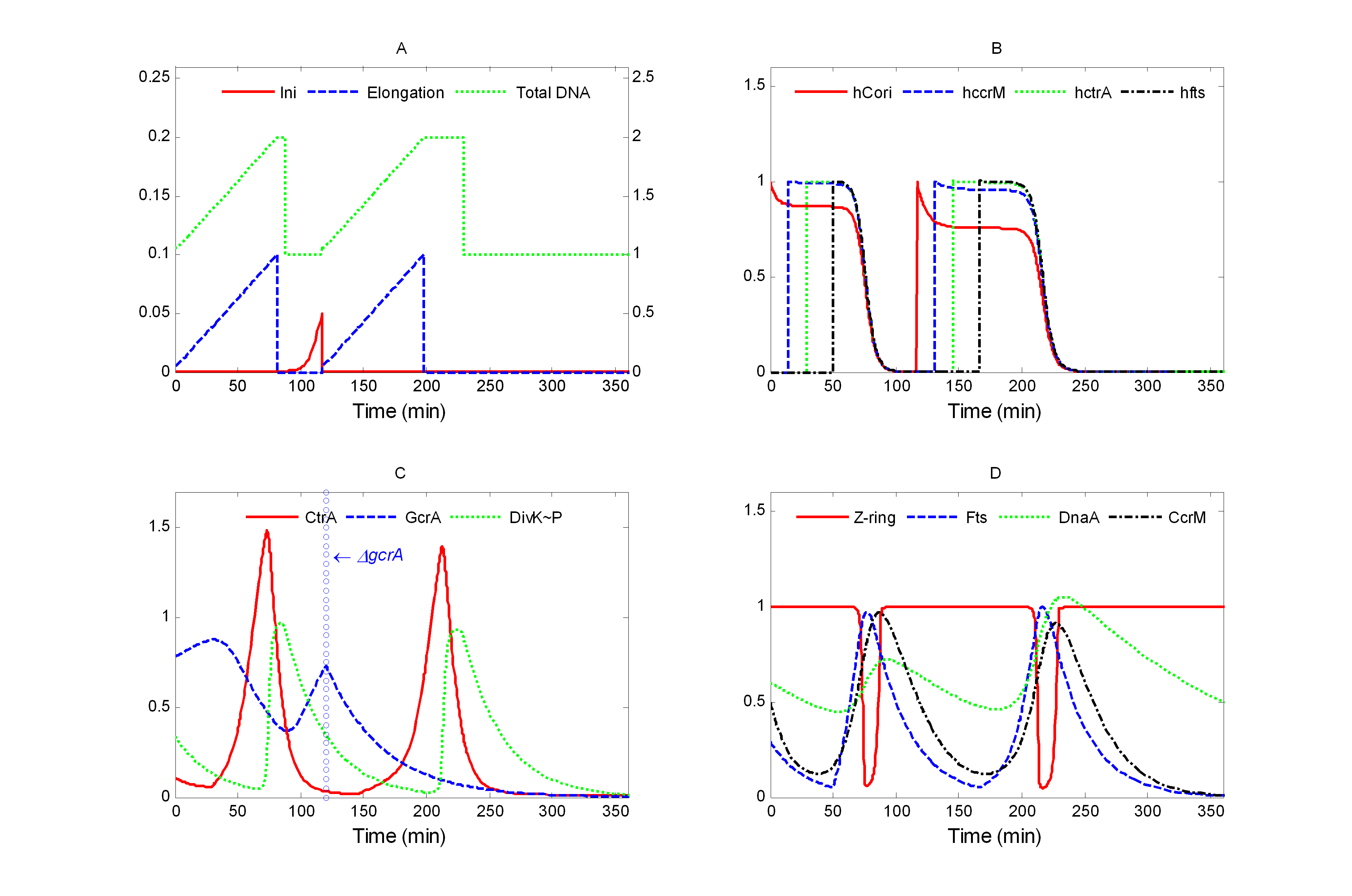

Supplement: Figure S5 — (563 KB TIF) [file pcbi.0040009.sg005.tif]

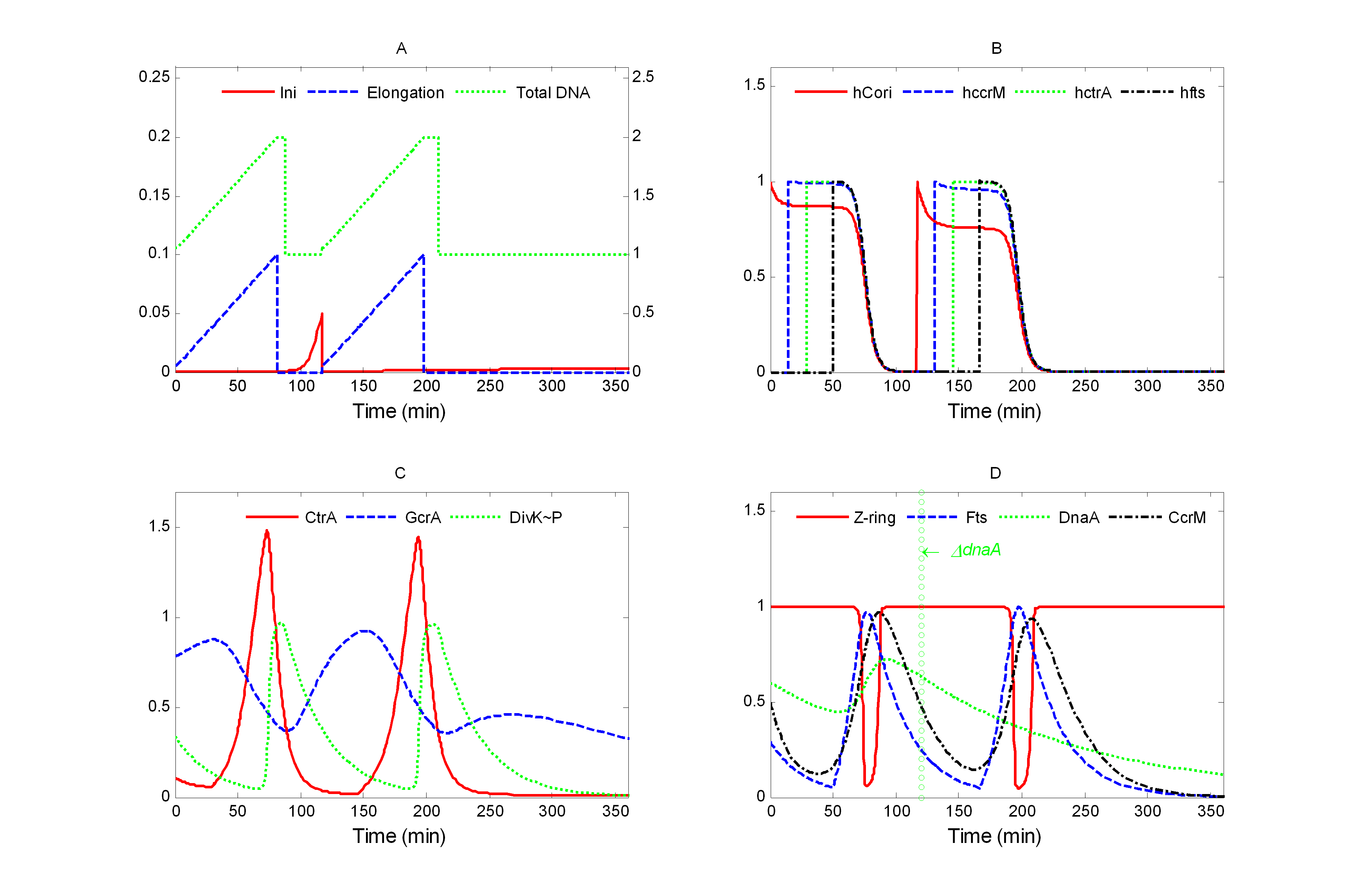

Supplement: Figure S6 — (564 KB TIF) [file pcbi.0040009.sg006.tif]

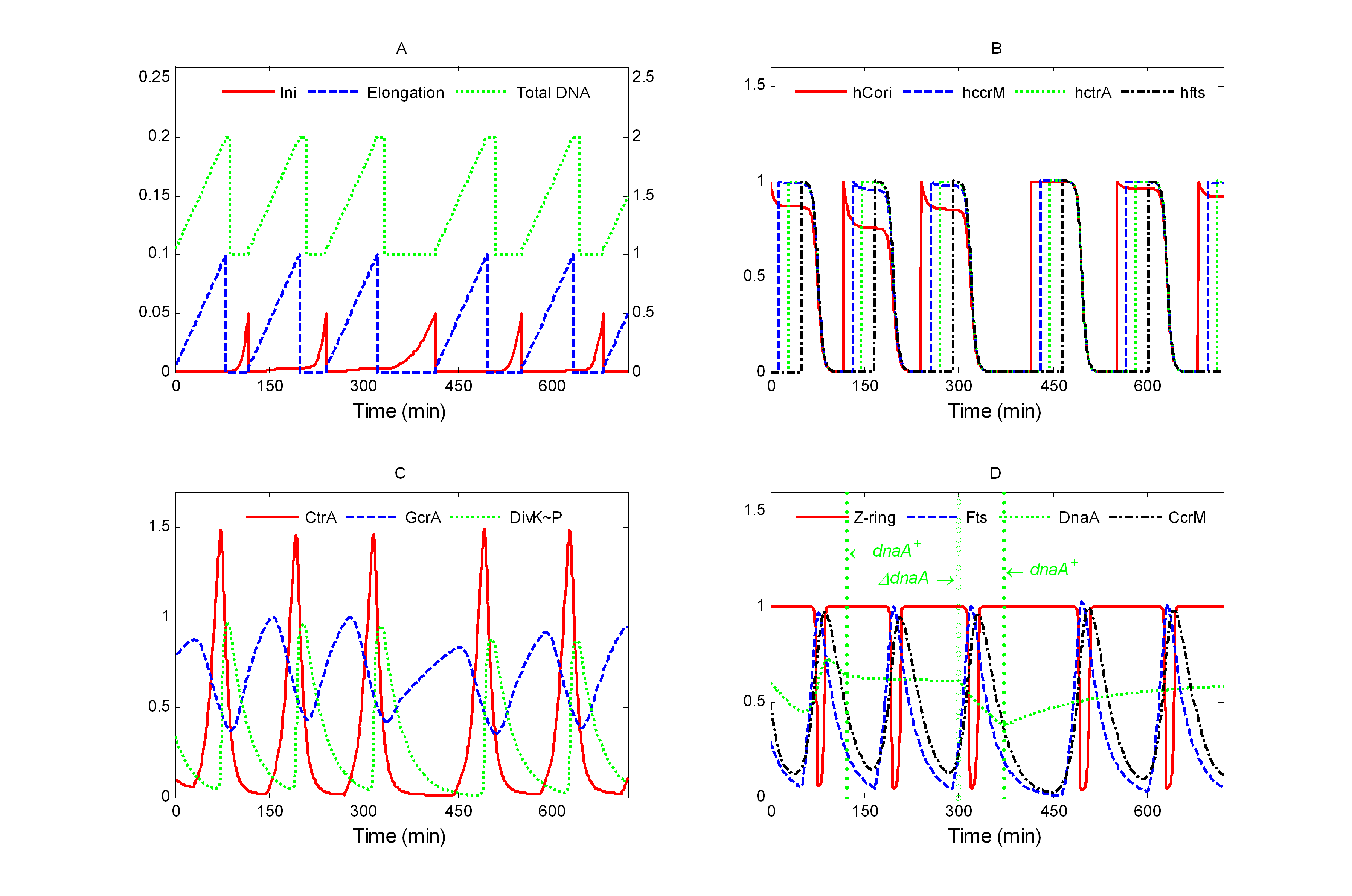

Supplement: Figure S7 — k s,DnaA = 0.0165 (WT) for 0 < t < 120 min, k s,DnaA = 0 and k ′ = 0.042 (constitutive expression from xylose-inducer promoter) for 120 < t < 300 min, k s,DnaA = 0 and k ′ = 0 for 300 < t < 370 min, and k s,DnaA = 0 and k ′ = 0.042 for t > 370 min. (623 KB TIF) [file pcbi.0040009.sg007.tif]

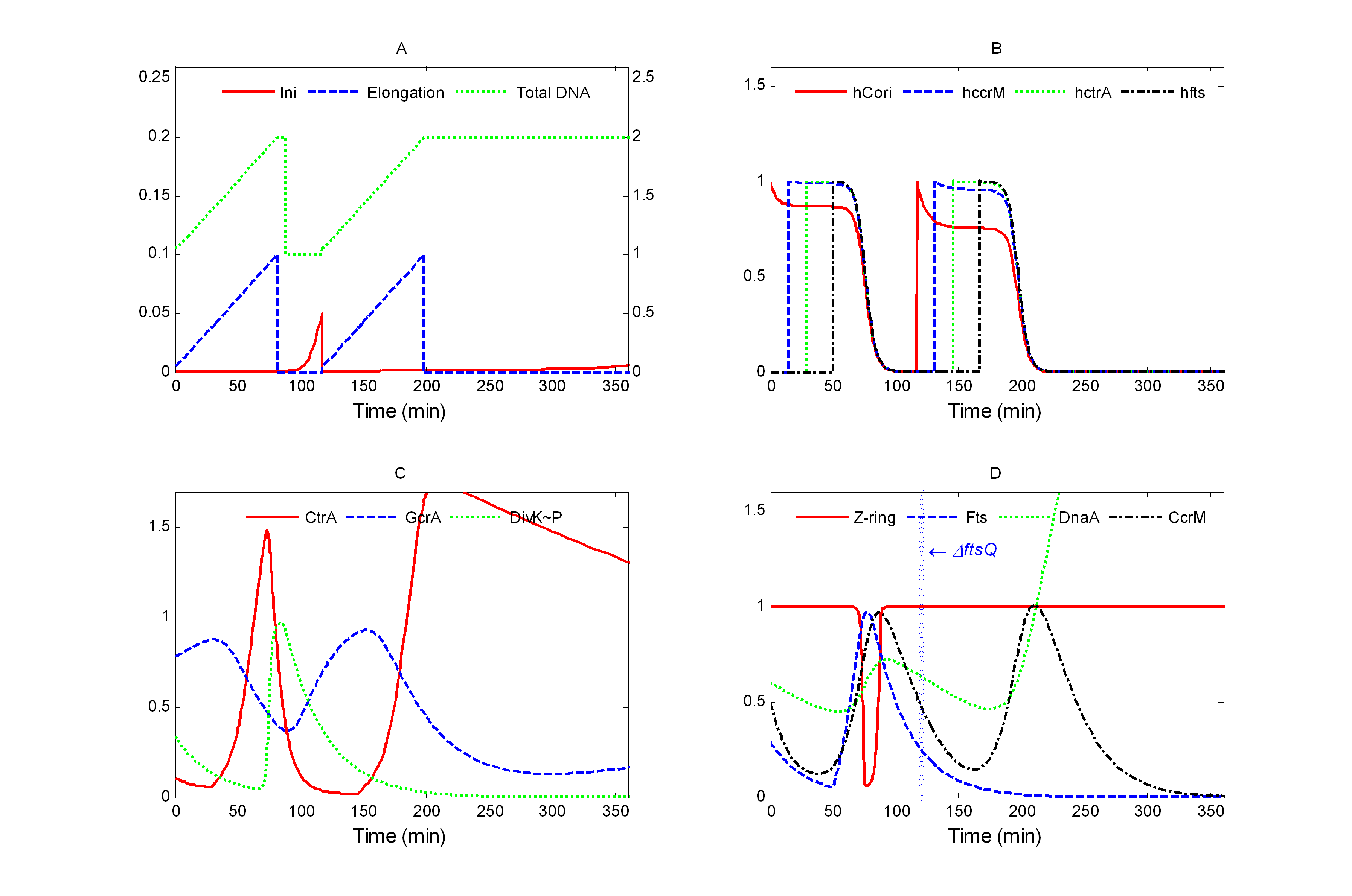

Supplement: Figure S8 — (558 KB TIF) [file pcbi.0040009.sg008.tif]

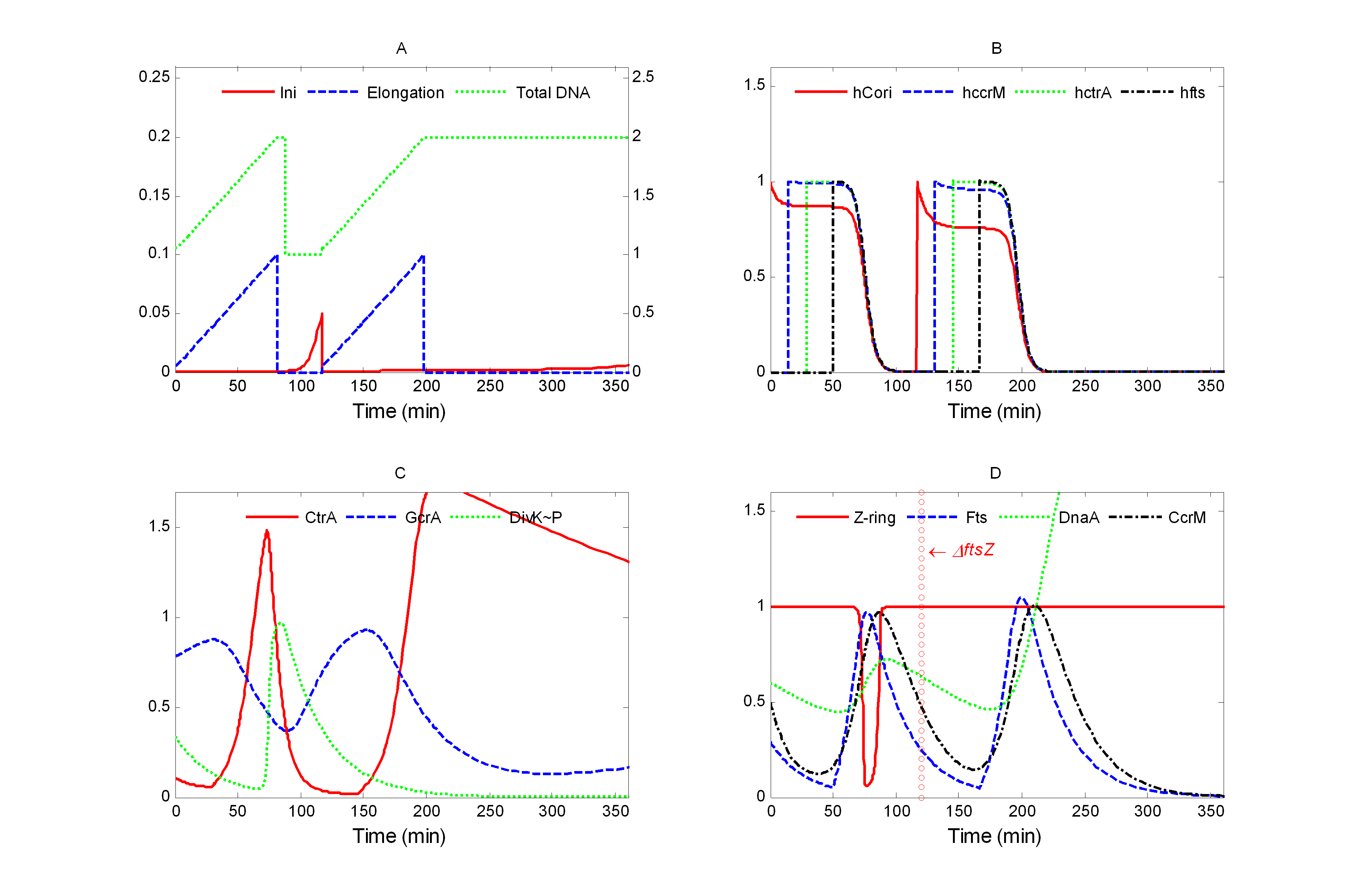

Supplement: Figure S9 — (560 KB TIF) [file pcbi.0040009.sg009.tif]

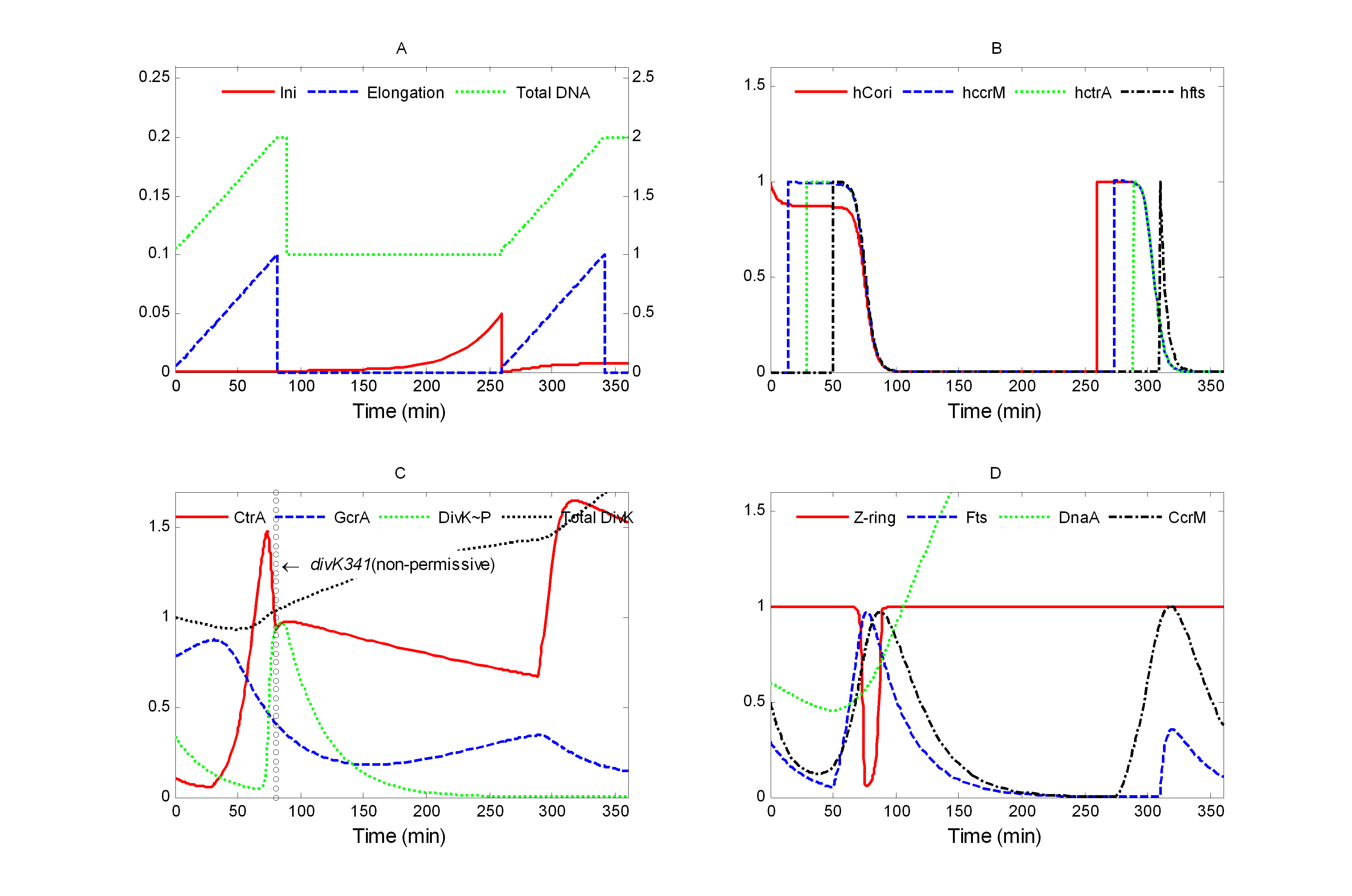

Supplement: Figure S10 — (561 KB TIF) [file pcbi.0040009.sg010.tif]

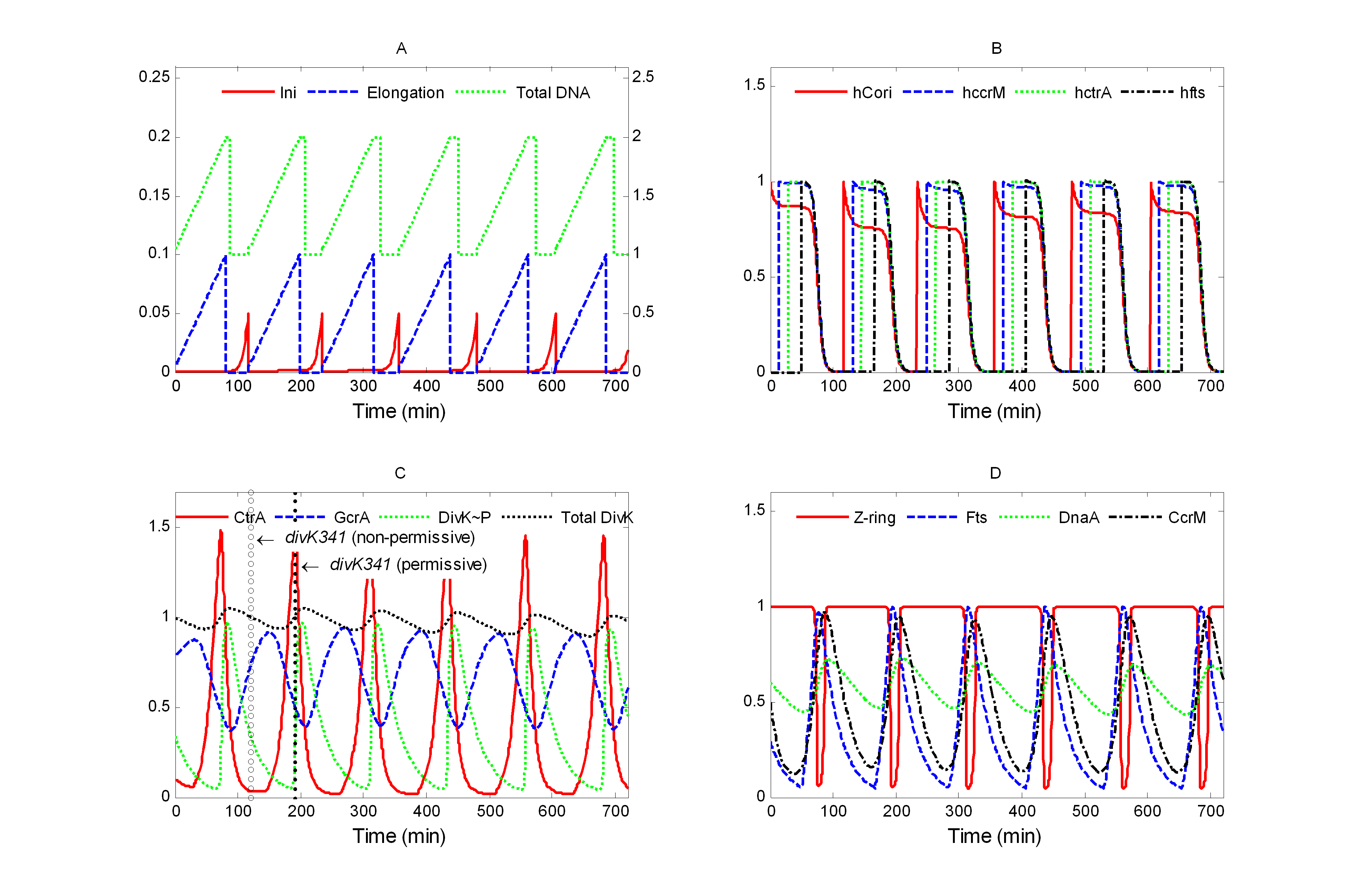

Supplement: Figure S11 — Shift to restrictive temperature (k d,ctrA2 = 0) at t = 120 min and back to permissive temperature (k d,ctrA2 = 0.15) at t = 190 min. (649 KB TIF) [file pcbi.0040009.sg011.tif]

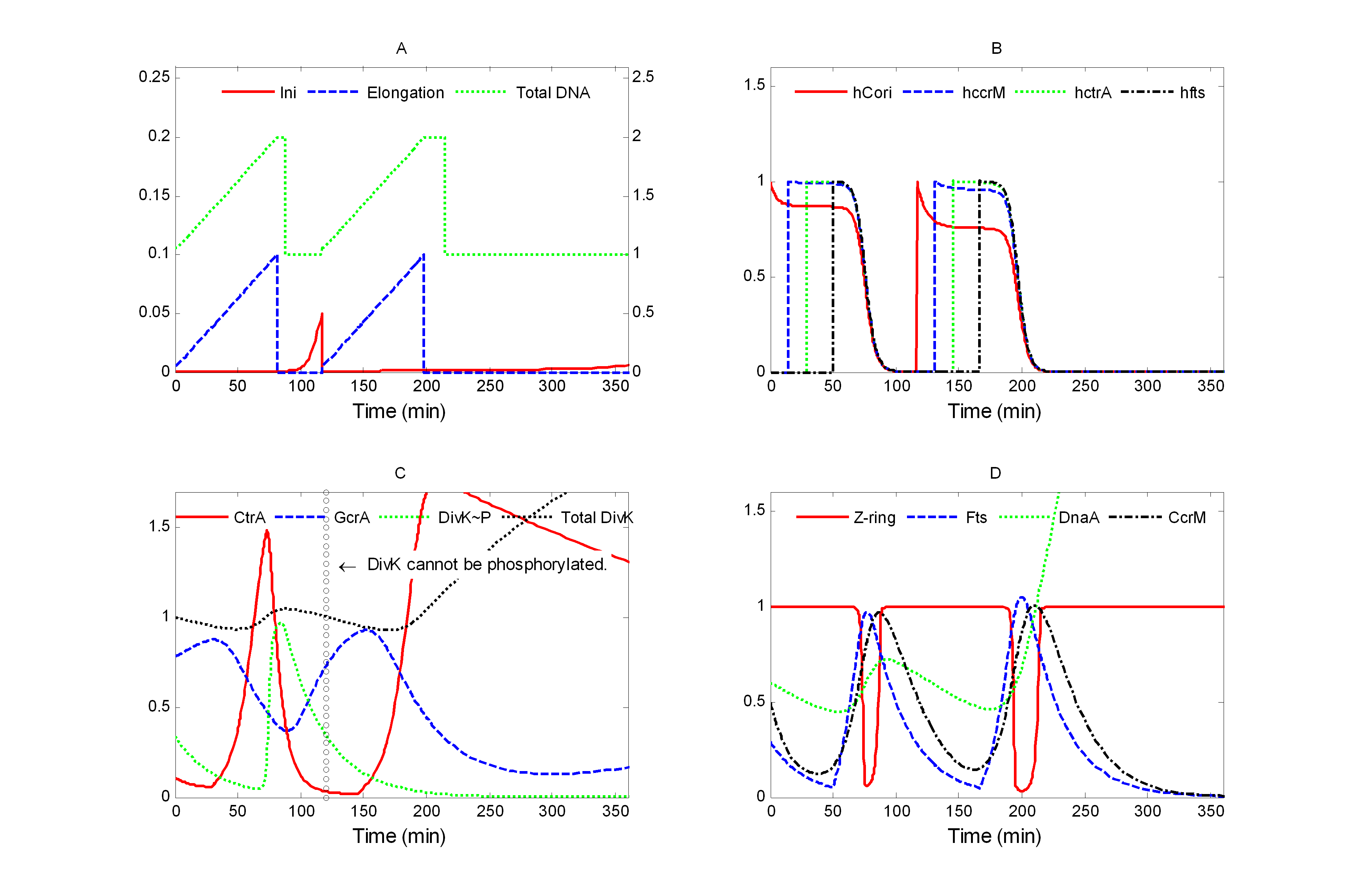

Supplement: Figure S12 — (570 KB TIF) [file pcbi.0040009.sg012.tif]

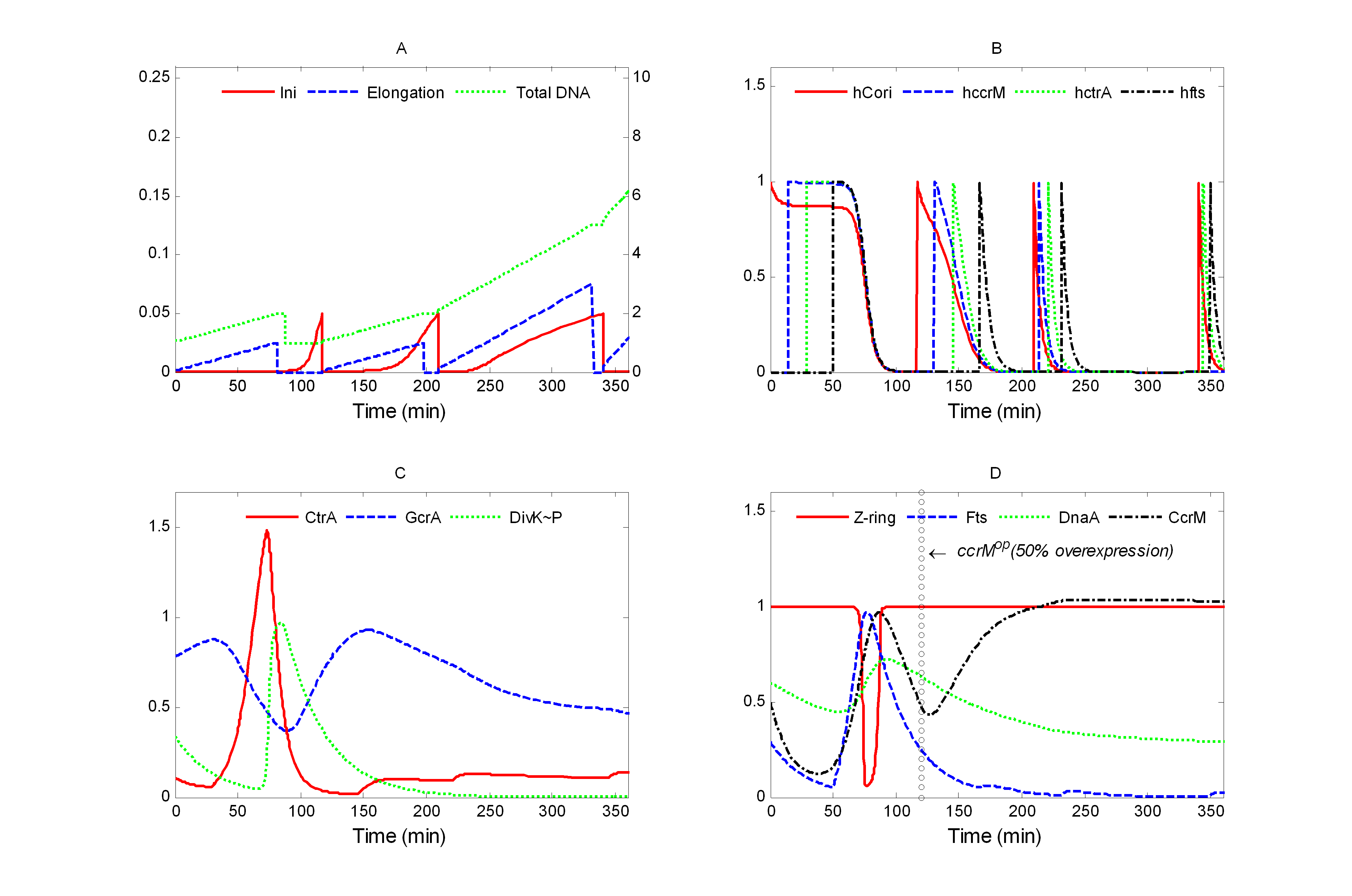

Supplement: Figure S13 — WT, wild-type. (569 KB TIF) [file pcbi.0040009.sg013.tif]

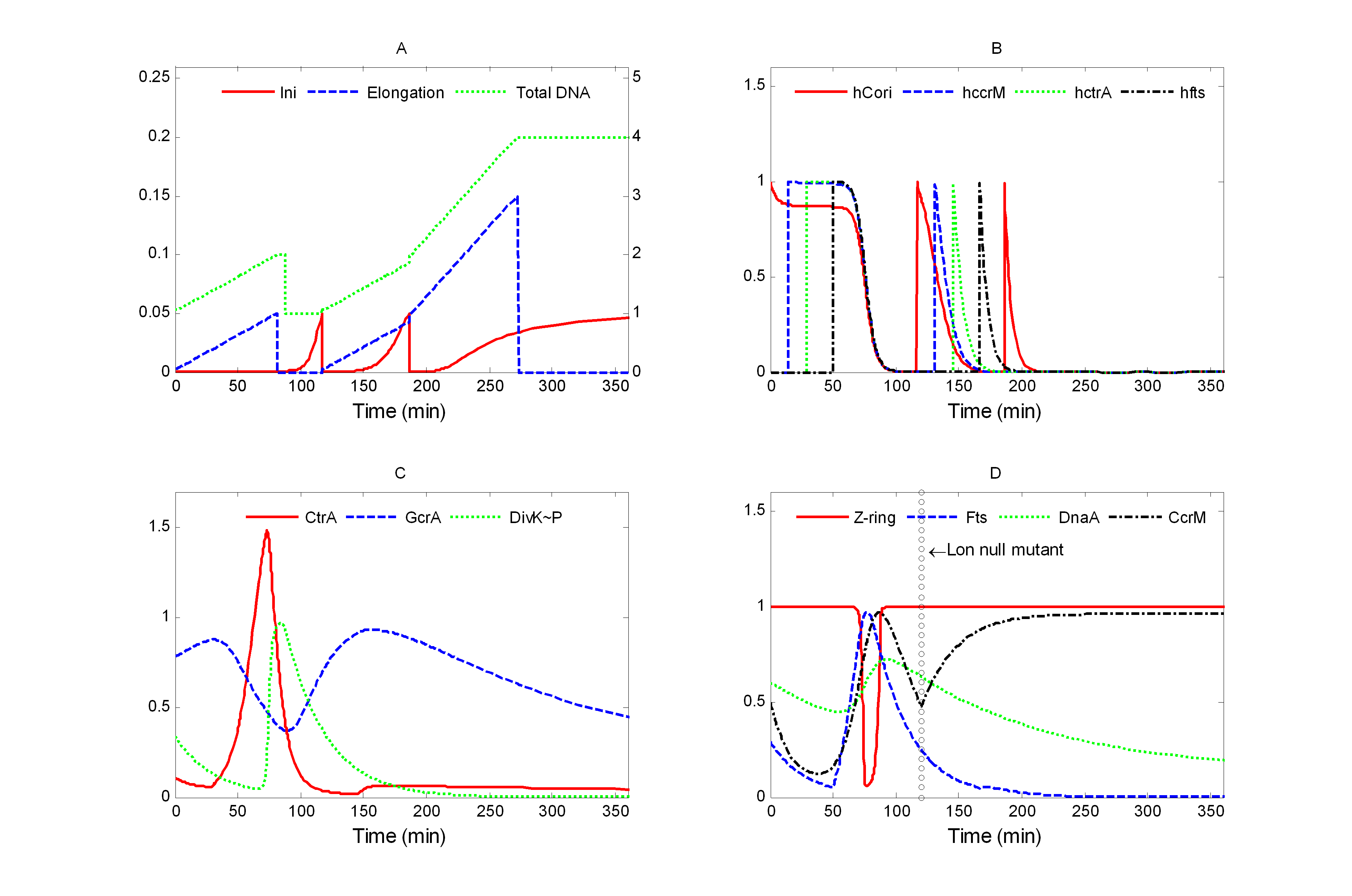

Supplement: Figure S14 — (555 KB TIF) [file pcbi.0040009.sg014.tif]

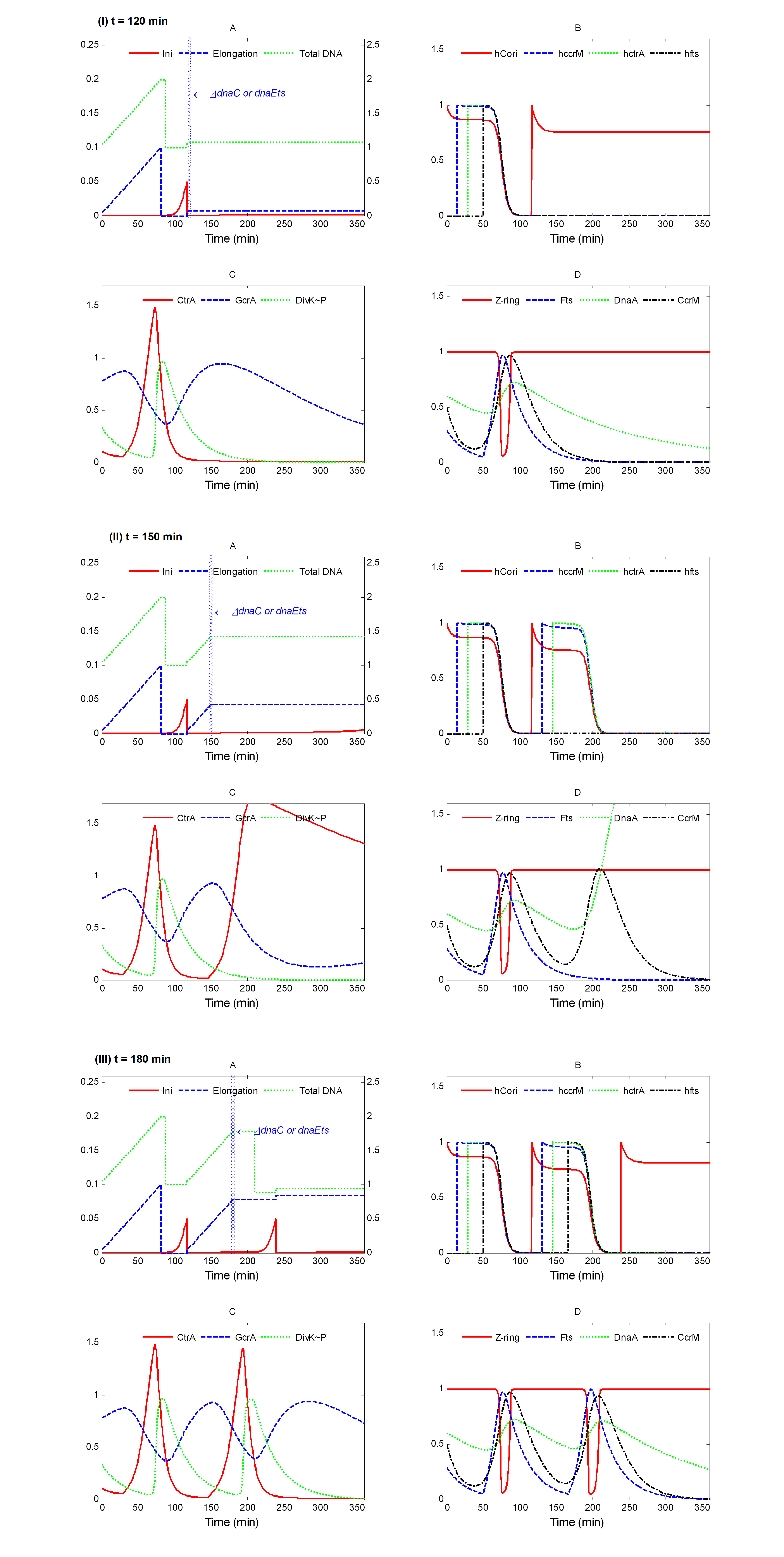

Supplement: Figure S15 — Mutation expressed at different times, as indicated. (1.6 MB TIF) [file pcbi.0040009.sg015.tif]

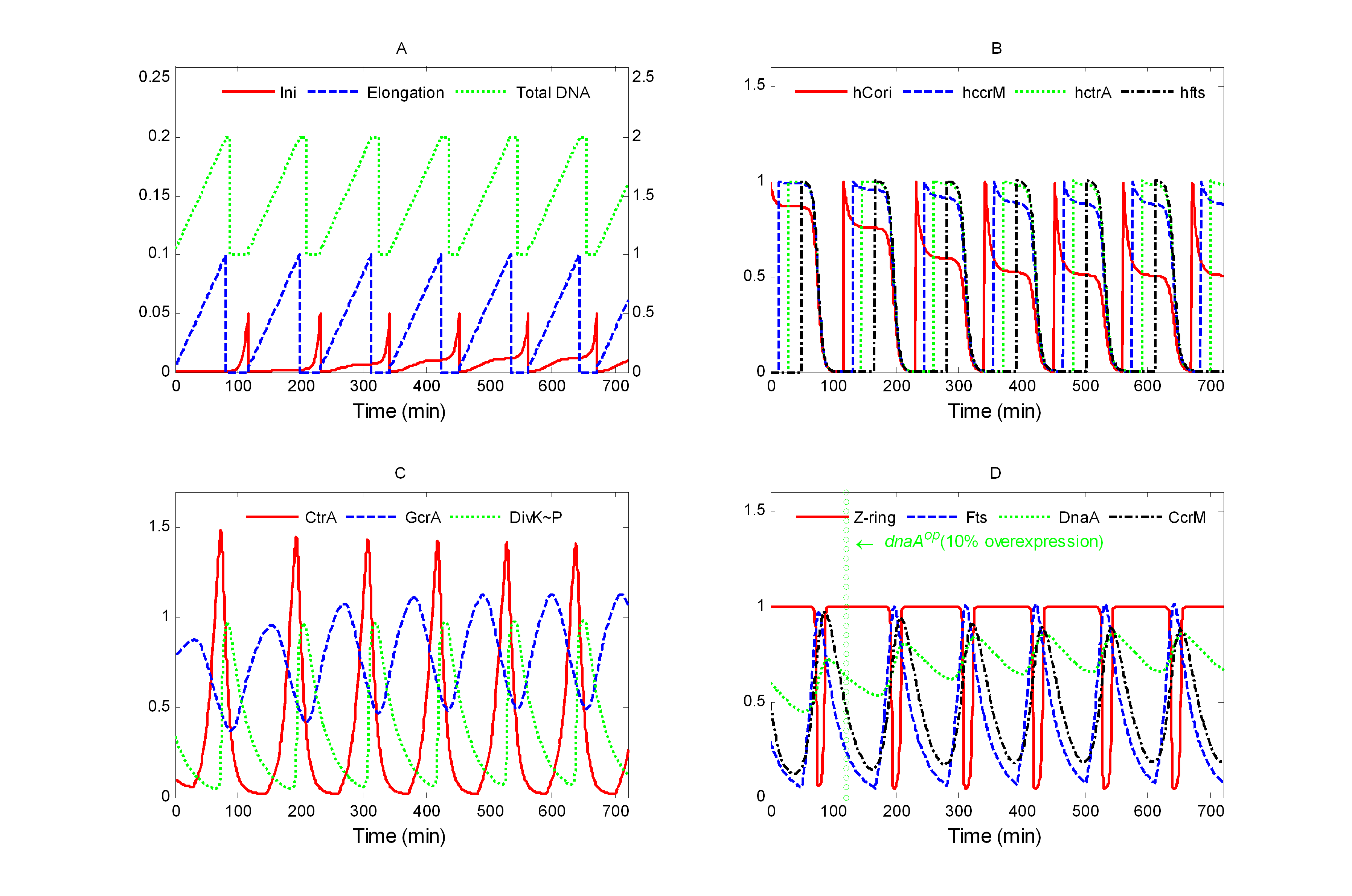

Supplement: Figure S17 — WT, wild-type. (649 KB TIF) [file pcbi.0040009.sg017.tif]
