# Supplementary material for: A Quantitative Study of the Division Cycle of Caulobacter crescentus Stalked Cells
Source: PLoS Comput Biol. 2008 Jan 25;4(1):e9. doi: 10.1371/journal.pcbi.0040009 (PMC2217572; doi:10.1371/journal.pcbi.0040009)
Supplement: Text S1 — (65 KB DOC) [file pcbi.0040009.sd001.doc]

**Online Supplementary Material**

**Supplementary Codes for Simulation**

**M-file for Matlab**

**Caulomodelwt.m**

**%&&&&&&&&&&&&&&&&&&&&&&&&&&&&&&&&&&&&&&&&&&&&&&&%**

**% Script for numericaly simulation of Caulobacter stalked cell cycle model %**

**% See the paper at the PLoS computational Biology' journal for details %**

**% Oct. 31, 2007. Shenghua Li, Paul Brazhnik, Bruno Sobral & John Tyson %**

**%&&&&&&&&&&&&&&&&&&&&&&&&&&&&&&&&&&&&&&&&&&&&&&&%**

**%**

**% This script needs 'eventsmodelwt.m' for running.**

**% To run the simulation, please input 'caulomodelwt' in the Matlab.**

**%&&&&&&&&&&&&&&&&&&&&&&&&&&&&&&&&&&&&&&&&&&&&&&&%**

**%**

**function caulo10312007 = caulomodelwt**

**clf;**

**%&&&&&&&&&&&&&&&&&&&&&&&&&&&&&&&&&&&&&&&&&&&&&&&%**

**%%variable map**

**%y(1)=[CtrA]**

**%y(2)=[GcrA]**

**%y(3)=[DnaA]**

**%y(4)=[Fts]**

**%y(5)=[Zring]**

**%y(6)=[DivK]**

**%y(7)=[DivK~P]**

**%y(8)=[Total DivK]**

**%y(9)=[I] (Intermediate)]**

**%y(10)=[CcrM]**

**%y(11)=[hCori] (hemimethylated Cori)**

**%y(12)=[hccrM] (hemimethylated ccrM)**

**%y(13)=[hctrA] (hemimethylated ctrA)**

**%y(14)=[hfts](hemimethylated fts)**

**%y(15)=[Ini] (Initiation)**

**%y(16)=[Elong](Elongation)**

**%y(17)=[DNA] (Total DNA)**

**%y(18)=Count (# of Chromosome)**

**%&&&&&&&&&&&&&&&&&&&&&&&&&&&&&&&&&&&&&&&&&&&&&&&%**

**%%Initial value of model variables, for a newborn, wild-type stalked cell**

**y0=zeros(18,1);**

**y0(1)=0.11;**

**y0(2)=0.78;**

**y0(3)=0.60;**

**y0(4)=0.29;**

**y0(5)=1.00;**

**y0(6)=0.66;**

**y0(7)=0.34;**

**y0(8)=1.0;**

**y0(9)=0.11;**

**y0(10)=0.5;**

**y0(11)=1;**

**y0(12)=0;**

**y0(13)=0;**

**y0(14)=0;**

**y0(15)=0.0;**

**y0(16)=0.05;**

**y0(17)=1.05;**

**y0(18)=2;**

**%%end of initial values**

**%&&&&&&&&&&&&&&&&&&&&&&&&&&&&&&&&&&&&&&&&&&&&&&&%**

**%%Integration parameters**

**tstart=0;**

**tfinal=360;**

**options=odeset('Events', @eventsmodelwt,'RelTol',1e-4,'AbsTol',1e-5);**

**%%end of integration parameters**

**%&&&&&&&&&&&&&&&&&&&&&&&&&&&&&&&&&&&&&&&&&&&&&&&&%**

**%%Numerical Integration**

**tout=tstart;**

**yout=y0.';**

**teout=[];**

**yeout=[];**

**ieout=[];**

**% loop for continous simulation of the differential equations.**

**while tstart < tfinal**

**[T,Y,TE,YE,IE]=ode15s(@modelwtin,[tstart tfinal],y0,options);**

**% Accumulate output.**

**nt=length(T);**

**tout=[tout;T(2:nt)];**

**yout=[yout;Y(2:nt,:)];**

**teout=[teout;TE];**

**yeout=[yeout;YE];**

**ieout=[ieout;IE];**

**% Refersh the initial conditions for differential equations once the interrupt**

**% point set in eventsmodelwt.m is detected.**

**y0=Y(nt,:);**

**if isscalar(IE)==0**

**IE=0;**

**end**

**switch (IE)**

**case 1**

**y0(16) = Y(nt, 16) + Y(nt, 18)*Y(nt, 15);**

**y0(17) = Y(nt, 17) + Y(nt, 18)*Y(nt, 15);**

**y0(18) = Y(nt, 18)*2;**

**y0(15) = 0;**

**y0(11) = 1;**

**case 2**

**y0(12)=Y(nt,12)+1;**

**case 3**

**y0(13)=Y(nt,13)+1;**

**case 4**

**y0(14)=Y(nt,14)+1;**

**case 5**

**y0(16)=0;**

**case 6**

**y0(17)=Y(nt,17)/2;**

**y0(18)=Y(nt,18)/2;**

**end**

**tstart=T(nt);**

**end**

**%%end of integration**

**%&&&&&&&&&&&&&&&&&&&&&&&&&&&&&&&&&&&&&&&&&&&&&&&%**

**%% Ploting the variables**

**figure(1);**

**set(gcf,'Name','Wild-type Simulation');**

**set(gcf,'outerposition', [-3 -3 1032 748]);**

**subplot(2,2,1)**

**p1=line(tout, yout(:,15), 'Color', 'r','LineWidth', 2, 'Linestyle', '-');**

**axis([0 360 0 0.26]);**

**ax1=gca;**

**set(ax1,'XTick',0:50:360, 'YTick', 0:0.05:0.26, 'Fontsize', 9);**

**ax2=axes('Position', get(ax1, 'Position'), 'YAxisLocation','right', 'Color', 'none', 'Box', 'on');**

**p2=line(tout, yout(:,16), 'Color','b', 'LineWidth', 2, 'Linestyle','--');**

**axis([0 360 0 2.6]);**

**set(ax2, 'YTick', 0:0.5:2.6, 'Fontsize', 9);**

**p3=line(tout, yout(:,17), 'Color','g', 'LineWidth', 2, 'Linestyle',':');**

**axis([0 360 0 2.6]);**

**xlabel('Time (min)','FontSize',11);**

**h = legend([p1, p2, p3], 'Ini', 'Elongation', 'Total DNA', 'Location', 'North');**

**set(h, 'Interpreter', 'none', 'Box', 'off', 'Orientation', 'horizontal');**

**title('A');**

**subplot(2,2,2)**

**p1=line(tout, yout(:,11), 'Color', 'r', 'LineWidth',2, 'Linestyle', '-');**

**p2=line(tout, yout(:,12), 'Color', 'b', 'LineWidth',2, 'Linestyle', '--');**

**p3=line(tout, yout(:,13), 'Color', 'g', 'LineWidth',2, 'Linestyle', ':');**

**p4=line(tout, yout(:,14), 'Color', 'k', 'LineWidth',2, 'Linestyle', '-.');**

**axis([0 360 0 1.6]);**

**ax1=gca;**

**set(ax1,'XTick',0:50:360,'YTick', 0:0.5:1.6, 'Fontsize', 9, 'Box', 'on');**

**xlabel('Time (min)','FontSize',11);**

**h = legend('hCori','hccrM','hctrA','hfts', 'Location', 'North');**

**set(h,'Interpreter','none','Box','off','Orientation','horizontal');**

**title('B');**

**subplot(2,2,3)**

**p1=line(tout, yout(:, 1), 'Color', 'r', 'LineWidth',2, 'Linestyle', '-');**

**p2=line(tout, yout(:, 2), 'Color', 'b', 'LineWidth',2, 'Linestyle', '--');**

**p3=line(tout, yout(:, 7), 'Color', 'g', 'LineWidth',2, 'Linestyle', ':');**

**axis([0 360 0 1.7]);**

**ax1=gca;**

**set(ax1,'XTick',0:50:360,'YTick', 0:0.5:1.7, 'Fontsize', 9, 'Box', 'on');**

**xlabel('Time (min)','FontSize',11);**

**h = legend('CtrA','GcrA','DivK~P', 'Location', 'North');**

**set(h,'Interpreter','none','Box','off','Orientation','horizontal');**

**title('C');**

**subplot(2,2,4)**

**p1=line(tout, yout(:, 5), 'Color', 'r', 'LineWidth',2, 'Linestyle', '-');**

**p2=line(tout, yout(:, 4), 'Color', 'b', 'LineWidth',2, 'Linestyle', '--');**

**p3=line(tout, yout(:, 3), 'Color', 'g', 'LineWidth',2, 'Linestyle', ':');**

**p4=line(tout, yout(:, 10), 'Color', 'k', 'LineWidth',2, 'Linestyle', '-.');**

**axis([0 360 0 1.6]);**

**ax1=gca;**

**set(ax1, 'XTick',0:50:360,'YTick', 0:0.5:1.6, 'Fontsize', 9, 'Box', 'on');**

**xlabel('Time (min)','FontSize',11);**

**h = legend('Z-ring', 'Fts', 'DnaA', 'CcrM', 'Location', 'North');**

**set(h,'Interpreter','none','Box','off','Orientation','horizontal');**

**title('D');**

**%end of ploting**

**%&&&&&&&&&&&&&&&&&&&&&&&&&&&&&&&&&&&&&&&&&&&&&&&%**

**%%&&&&&&&&&&&&&&&&&&&&&&&&&&&&&&&&&&&&&&&&&&&&&&%**

**% The input equations of model for wild-type cells %**

**%&&&&&&&&&&&&&&&&&&&&&&&&&&&&&&&&&&&&&&&&&&&&&&&%**

**function dydt=modelwtin(t, y)**

**%&&&&&&&&&&&&&&&&&&&&&&&&&&&&&&&&&&&&&&&&&&&&&&&%**

**%%Parameters values for the equations**

**ksCtrAP1=0.0083; JiCtrACtrA=0.4; niCtrACtrA=2;**

**ksCtrAP2=0.073; JaCtrACtrA=0.45; naCtrACtrA=2;**

**kdCtrA1=0.002;**

**kdCtrA2=0.15; ndCtrA2=2;**

**JdCtrADivKP=0.55;**

**ksGcrA=0.045; JiGcrACtrA=0.2; niGcrACtrA=2;**

**kdGcrA=0.022;**

**ksFts=0.063; kdFts=0.035;**

**kzringopen=0.8; Jaopen=0.01;**

**kzringclosed1=0.0001; Jaclosed1=0.1;**

**kzringclosed2=0.6; nzringclosed2=4;**

**JZringFts=0.78;**

**ksDivK=0.0054; ktransDivKP=0.0295; ktransDivK=0.5; kdDivK=0.002;**

**ksI=0.08; kdI=0.04;**

**ksCcrM=0.072; kdCcrM=0.07;**

**kaDnaA=0.0165; JiDnaAGcrA=0.5; niDnaAGcrA=2;**

**kdDnaA=0.007;**

**kaIni=0.01; JaIni=1; naIni=4;**

**thetaCtrA=0.2; nthetaCtrA=4;**

**thetaDnaA=0.6; nthetaDnaA=4;**

**thetaGcrA=0.45; nthetaGcrA=4;**

**thetaCori=0.0002; nthetaCori=1;**

**kmcori=0.4; Jmcori=0.95; nmcori=4;**

**kmccrM=0.4; JmccrM=0.95; nmccrM=4;**

**kmctrA=0.4; JmctrA=0.95; nmctrA=4;**

**kmfts=0.4; Jmfts=0.95; nmfts=4;**

**kelong=0.95/160;**

**nelong=4;**

**%$end of parameters**

**%&&&&&&&&&&&&&&&&&&&&&&&&&&&&&&&&&&&&&&&&&&&&&&&%**

**%%Differential equations of the model**

**dydt=zeros(18,1);**

**dydt(1)=(ksCtrAP1*JiCtrACtrA^niCtrACtrA/(JiCtrACtrA^niCtrACtrA+y(1)^niCtrACtrA)*y(2)+ksCtrAP2*y(1)^naCtrACtrA/(JaCtrACtrA^naCtrACtrA+y(1)^naCtrACtrA))*y(13)-(kdCtrA1+kdCtrA2*y(7)^ndCtrA2/(JdCtrADivKP^ndCtrA2+y(7)^ndCtrA2))*y(1);**

**dydt(2)=(ksGcrA*JiGcrACtrA^niGcrACtrA/(JiGcrACtrA^niGcrACtrA+y(1)^niGcrACtrA)*y(3)-kdGcrA*y(2));**

**dydt(3)=kaDnaA*JiDnaAGcrA^niDnaAGcrA/(JiDnaAGcrA^niDnaAGcrA+y(2)^niDnaAGcrA)*y(1)*(2-y(11))-kdDnaA*y(3);**

**dydt(4)=ksFts*y(1)*y(14)-kdFts*y(4);**

**dydt(5)=(kzringopen*(1-y(5))/(0.01+(1-y(5)))-(kzringclosed1+kzringclosed2*(y(4)/JZringFts)^nzringclosed2)*y(5)/(0.05+y(5)));**

**dydt(6)=(ksDivK*y(1)+ktransDivKP*y(7)-ktransDivK*(1-y(5))*y(6)-kdDivK*y(6));**

**dydt(7)=(-ktransDivKP*y(7)+ktransDivK*(1-y(5))*y(6)-kdDivK*y(7));**

**dydt(8)=(ksDivK*y(1)-kdDivK*y(8));**

**dydt(9)=ksI*y(12)*y(1)-kdI*y(9);**

**dydt(10)=ksCcrM*y(9)-kdCcrM*y(10);**

**dydt(11)=-kmcori*y(10)^nmcori/(Jmcori^nmcori+y(10)^nmcori)*y(11);**

**dydt(12)=-kmccrM*y(10)^nmccrM/(JmccrM^nmccrM+y(10)^nmccrM)*y(12);**

**dydt(13)=-kmctrA*y(10)^nmctrA/(JmctrA^nmctrA+y(10)^nmctrA)*y(13);**

**dydt(14)=-kmfts*y(10)^nmfts/(Jmfts^nmfts+y(10)^nmfts)*y(14);**

**dydt(15)=kaIni*(y(3)/thetaDnaA)^nthetaDnaA*(y(2)/thetaGcrA)^4/(JaIni^naIni+(y(1)/thetaCtrA)^nthetaCtrA+(y(3)/thetaDnaA)^nthetaDnaA+(y(2)/thetaGcrA)^nthetaGcrA+(y(11)/thetaCori)^nthetaCori);**

**dydt(16)=kelong*y(16)^nelong/(y(16)^nelong+0.05^nelong)*y(18);**

**dydt(17)=kelong*y(16)^nelong/(y(16)^nelong+0.05^nelong)*y(18);**

**dydt(18)=0;**

**%%end of equations**

**%&&&&&&&&&&&&&&&&&&&&&&&&&&&&&&&&&&&&&&&&&&&&&&&%**

**%%Clear the Computing**

**end**

**clc;**

**clear all;**

**end**

**%&&&&&&&&&&&&&&&&&&&&&&&&&&&&&&&&&&&&&&&&&&&&&&&%**

**Eventsmodelwt.m**

**%&&&&&&&&&&&&&&&&&&&&&&&&&&&&&&&&&&&&&&&&&&&&&&&%**

**% Script for numericaly simulation of Caulobacter stalked cell cycle model %**

**% See the paper at the PLoS computational Biology' journal for details %**

**% Oct. 31, 2007. Shenghua Li, Paul Brazhnik, Bruno Sobral & John Tyson %**

**%&&&&&&&&&&&&&&&&&&&&&&&&&&&&&&&&&&&&&&&&&&&&&&&%**

**%**

**% This is the script for detecting specific events of stalked cell cycle.**

**% This script is required by 'caulomodelwt.m' for running.**

**%&&&&&&&&&&&&&&&&&&&&&&&&&&&&&&&&&&&&&&&&&&&&&&&%**

**function [value,isterminal,direction] = eventsmodewt(t, y)**

**%&&&&&&&&&&&&&&&&&&&&&&&&&&&&&&&&&&&&&&&&&&&&&&&%**

**% Locate the time when values passes through zero in a increasing direction**

**% and stop integration.**

**value=[sign(y(15)-0.05);sign(y(16)-0.2);sign(y(16)-0.375);sign(y(16)-0.625);sign(y(16)+1-y(18));sign(y(5)-0.9)];**

**isterminal=[1;1;1;1;1;1];**

**direction=[+1;+1;+1;+1;+1;+1];**

**%&&&&&&&&&&&&&&&&&&&&&&&&&&&&&&&&&&&&&&&&&&&&&&&%**

**end**

**%&&&&&&&&&&&&&&&&&&&&&&&&&&&&&&&&&&&&&&&&&&&&&&&%**
