# Supplementary material for: A Quantitative Study of the Division Cycle of Caulobacter crescentus Stalked Cells
Source: PLoS Comput Biol. 2008 Jan 25;4(1):e9. doi: 10.1371/journal.pcbi.0040009 (PMC2217572; doi:10.1371/journal.pcbi.0040009)
Supplement: Text S2 — (46 KB DOC) [file pcbi.0040009.sd002.doc]

**Online Supplementary Material**

**Genes and proteins that appear in our manuscript**

|  | | |  |  |  |
| --- | --- | --- | --- | --- | --- |
| **CC#** | **GenBankName** | **GenBank Annotation** | **GenBankID** | **TIGR ORF #** | **EC #** |
| CC0008 | dnaA | chromosomal replication initiator protein DnaA | 13421094 | ORF02307 |  |
| CC0378 | ccrM | modification methylase CcrMI | 13421535 | ORF03051 | 2.1.1.72 |
| CC0744 | cpdR | response regulator | 13421977 | ORF03749 |  |
| CC1063 | divJ | sensor histidine kinase DivJ | 13422363 | ORF04405 |  |
| CC1078 | cckA | cell cycle histidine kinase CckA | 13422380 | ORF04430 |  |
| CC1307 | perP | conserved hypothetical protein | 13422646 | ORF04859 |  |
| CC1960 | lon | ATP-dependent protease LA | 13423421 | ORF06080 | 3.4.21.53 |
| CC1961 | clpX | ATP-dependent Clp protease, ATP-binding subunit ClpX | 13423422 | ORF06082 |  |
| CC1963 | clpP | ATP-dependent Clp protease, proteolytic subunit | 13423424 | ORF06085 | 3.4.21.92 |
| CC2045 | podJ | polar organelle development protein, authentic frameshift |  | ORF06244 |  |
| CC2245 | gcrA | hypothetical protein | 13423754 | ORF06648 |  |
| CC2463 | divK | polar differentiation response regulator | 13424012 | ORF07061 |  |
| CC2482 | pleC | non-motile and phage-resistance protein | 13424035 | ORF07098 |  |
| CC2540 | ftsZ | cell division protein FtsZ | 13424103 | ORF07201 |  |
| CC2541 |  | cell division protein FtsA | 13424104 | ORF07204 |  |
| CC2542 | ftsQ | cell division protein FtsQ | 13424105 | ORF07205 |  |
| CC3035 | ctrA | cell cycle transcriptional regulator CtrA | 13424677 | ORF00839 |  |
| CC3295 | rcdA | hypothetical protein | 13424989 | ORF01381 |  |
| This table is derived from (Laub et al., 2000). | | |  |  |  |
